# Development and Validation of a Novel Prognostic Model for Lower-Grade Glioma Based on Enhancer RNA-Regulated Prognostic Genes

**DOI:** 10.3389/fonc.2022.714338

**Published:** 2022-03-01

**Authors:** Wei Tian, Guangcan Yan, Kegong Chen, Xinhao Han, Wei Zhang, Lin Sun, Qi Zhang, Yafeng Zhang, Yan Li, Meina Liu, Qiuju Zhang

**Affiliations:** ^1^ Department of Biostatistics, School of Public Health, Harbin Medical University, Harbin, China; ^2^ Department of Cardio-Thoracic Surgery, The Second Affiliated Hospital of Harbin Medical University, Harbin, China; ^3^ Department of Health Management, School of Health Management, Harbin Medical University, Harbin, China

**Keywords:** enhancer RNA, super-enhancer RNA, glioma, prognosis, nomogram

## Abstract

Enhancer RNAs (eRNAs) are present specifically in tumors, where they affect the expression of eRNA-regulated genes (ERGs). Owing to this characteristic, ERGs were hypothesized to improve prognosis of overall survival in heterogeneous low-grade and intermediate-grade gliomas. This study aimed to construct and validate an ERG prognostic tool to facilitate clinical management, and offer more effective diagnostic and therapeutic biomarkers for glioma. Survival-related eRNAs were identified, and their ERGs were selected based on eRNA and target gene information. The ERG prognostic model was constructed and validated using internal and external validation cohorts. Finally, biological differences related to the ERG signature were analysed to explore the potential mechanisms influencing survival outcomes. Thirteen ERGs were identified and used to build an ERG risk signature, which included five super-enhancer RNA (seRNA)-regulated genes and five LGG-specific eRNA-regulated genes. The prognostic nomogram established based on combining the ERG score, age, and sex was evaluated by calibration curves, clinical utility, Harrell’s concordance index (0.86; 95% CI: 0.83-0.90), and time-dependent receiver operator characteristic curves. We also explored potential immune-related mechanisms that might cause variation in survival. The established prognostic model displayed high validity and robustness. Several immune-related genes regulated by seRNAs or specific eRNAs were identified, indicating that these transcripts or their genes were potential targets for improving immunotherapeutic/therapeutic outcomes. The functions of an important specific eRNA-regulated gene (USP28) were validated in robust vitro experiments. In addition, the ERG risk signature was significantly associated with the immune microenvironment and other immune-related features.

## Introduction

Gliomas, derived from glial cells or glial precursor cells, are the most common lethal primary tumors of the central nervous system (1). Traditionally, malignant gliomas are divided into three categories: low-grade, intermediate-grade, and high-grade gliomas. The first two types, known as lower-grade gliomas (LGGs), account for approximately 43.2% of all gliomas and are relatively slow-growing but prone to recurrence (1, 2). Therefore, they have greater therapeutic and public health value than high-grade gliomas, which lead to worse outcomes and a median overall survival (OS) of < 14.4 months (3). Despite advances in treatment regimens, therapeutic outcomes remain poor and with wide variations in OS (4, 5). To improve the effectiveness of treatment and postoperative management, OS predictions should be more accurate; however, this depends on the availability of precise biomarkers and prognostic tools. Several genetic biomarkers have been identified as prognostic tools for LGG outcomes, yet their precision and specificity are limited by the strong heterogeneity of LGGs (6–8). Therefore, to predict outcomes and improve treatment quality, more sensitive and specific biomarkers are required.

As important distal regulatory DNA elements, enhancers are direct drivers of carcinogenesis. Enhancer RNAs (eRNAs) represent functional active enhancers, which affect OS in tumors by interacting with transcription factors, cofactors, and RNA-binding proteins; trapping transcription factors; and modulating the process of RNA Pol II pause-release (9, 10). More importantly, their robustly specific expression across different tissues makes them effective and powerful diagnostic and/or prognostic biomarkers (11). Specific eRNAs have been shown to correlate highly with numerous genes participating in tumor signaling pathways, thus confirming their importance in regulating tumor onset, progression, and prognosis (11). Super-enhancers, whose transcripts are termed super-enhancer RNAs (seRNAs), comprise more than one enhancer in series. They could recruit 10-times more regulators to affect the activity of target eRNA-regulated genes (ERGs) (12, 13). Whereas previous studies have focused extensively on the relationship between eRNAs and ERGs, it remains to be determined whether ERGs could be used as effective biomarkers to predict survival outcomes in patients with LGGs.

The purpose of this study was to identify prognostic genes regulated by eRNAs/seRNAs through integration of eRNA and mRNA expression data, and to establish a novel ERG prognostic model. Furthermore, potential biological mechanisms related to ERGs and diagnostic/therapeutic targets were explored with the intent of ameliorating individualized treatment and ultimately improving outcomes for LGGs patients.

## Material and Methods

### Dataset Selection

Five datasets were employed in this study. The first included eRNA data from the database of enhancer RNA in cancers (eRic; https://hanlab.uth.edu/eRic/), which was used to identify survival-related eRNAs and obtain information about regulatory interactions between eRNAs and ERGs. The second included gene expression and clinical data from the UCSC Cancer Browser (TCGA; https://xena.ucsc.edu/), which was used to establish the prognostic model and served as the internal validation set. The third dataset was from the Chinese Glioma Genome Atlas (CGGA; http://cgga.org.cn/), which served as the external validation dataset. The fourth one comprised immune infiltration data, obtained from the Tumor Immune Estimation Resource (TIMER; http://cistrome.org/TIMER) and was used to explore the relationship between ERGs and immune infiltration. The last one was the copy number variation data downloaded from the cBioPortal interface (cBioPortal; https://www.cbioportal.org/).

### Inclusion Criteria

**Figure 1:**
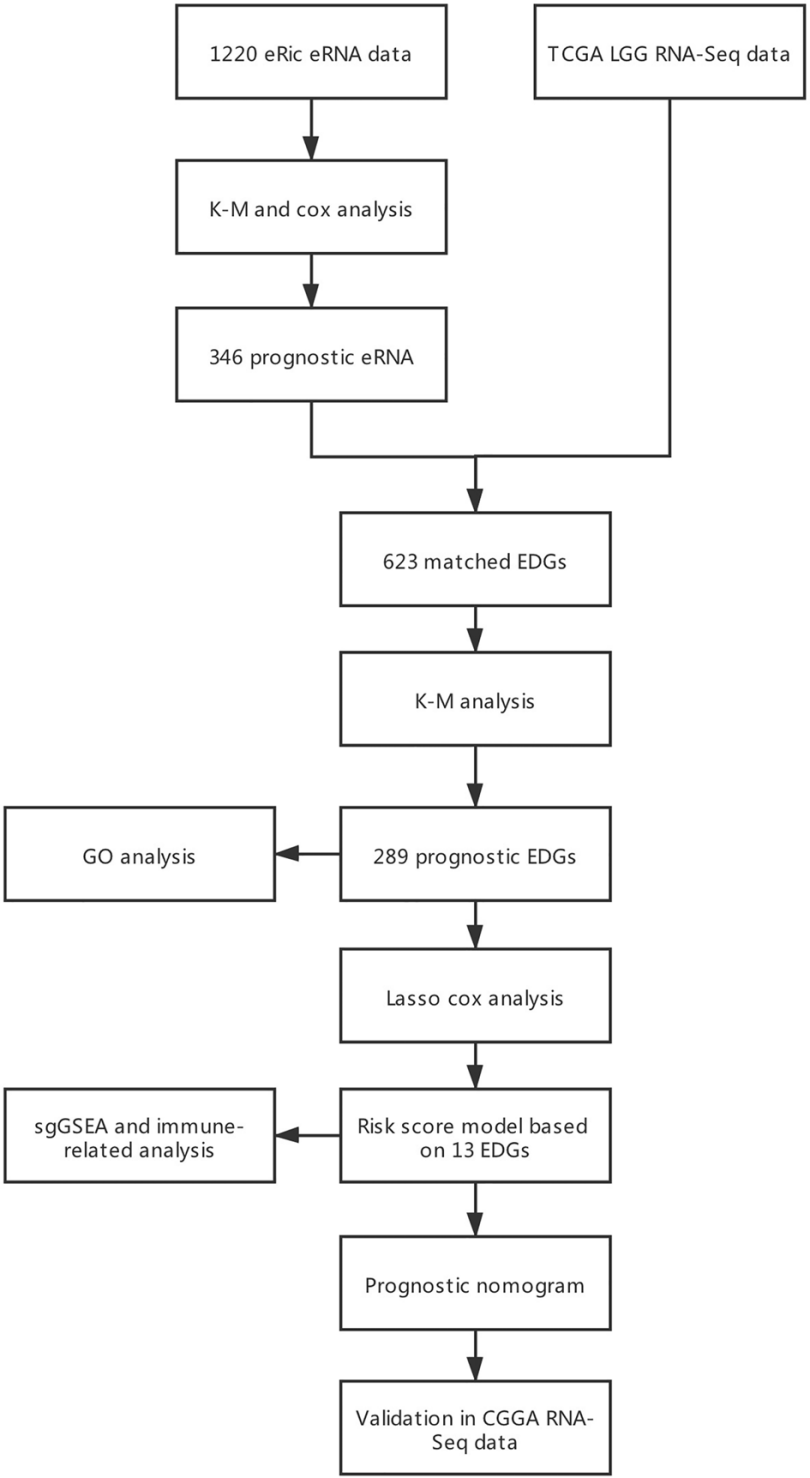
Flowchart of developing and validating the EDGs prognostic nomogram.

Inclusion criteria were as follows: (1) primary glioma, (2) histopathological diagnosis confirmed as WHO grade II or III, and (3) OS > 30 days (**Figure 1**). In the training and external validation datasets, LGG patients with missing clinical and survival information were excluded if the incomplete pattern was missing completely at random (MCAR) (14). Batch effects in expression data were corrected by the ComBat method in the external CGGA cohort, which contained two datasets with batch effects.

### Identification of Prognostic eRNAs, Their ERGs, and Construction of the ERG Signature

Prognostic eRNAs were identified after Kaplan-Meier and univariate Cox analyses, with adjusted *p* < 0.05 as the cut-off criterion. Regulatory relationships between eRNAs and their potential ERGs were obtained from previous studies (11). Kaplan-Meier analysis was used to find the potential important survival-related ERGs.

The most significant prognostic ERGs were selected by the LASSO-Cox model and were used to establish a predictive tool in case of a strong correlation between ERGs and their eRNAs (correlation coefficient *r_s_* > 0.3, *p* < 0.05). The risk score was calculated based on ERG expression levels and their relative coefficients in the LASSO-Cox model, and then classified into three grades based on the tertiles of the score.

Gene Ontology (GO) enrichment analysis was performed to explore the biological functions of candidate ERGs using the *clusterProfiler* package in R. This allowed verification of the relationship between survival-related ERGs and gliomas.

### Development and Assessment of the ERG Prognostic Model

Considering jointly the ERG signature and traditional clinical variables, univariate and multivariate Cox models were employed to select reliable prognostic features with which to construct the predictive model. In multivariate Cox analysis, a stepwise process was used to confirm crucial characteristics and the proportional hazards assumption test was performed to the final prognostic model. To visualize the prognostic tool and facilitate it to predict survival outcomes, the established model was transformed into a nomogram that could intuitively and help individuals with certain factors to determine an instantaneous failure rate at a given time. In fact, the failure rate was calculated by the underlying model we had established before (15).

As promising biomarkers, the telomerase reverse transcriptase promoter (TERTp) mutation and O-6-methylguanine-DNA-methyltransferase (MGMT) promoter methylation were important biomarkers that were all related to OS. However, they were not easy to obtain directly from the TCGA cohort (16). To incorporate them into consideration, we used the MGMT methylation level to represent the MGMT promoter methylation status, and the TERT gene mRNA expression level to estimate the TERTp mutation status, which was mainly based on the high correlation between methylation level and methylation status for MGMT and between gene expression level and promoter mutate for TERT, as previous researches described (17). And the relationship between MGMT methylation and IDH status was analyzed to explore their potential mutual effect in the prognostic model.

To establish a reliable prognostic model, it is necessary to evaluate its different aspects, including discriminative ability, accuracy, and clinical utility. In the development cohort, Harrell’s concordance index (C-index) was used to comprehensively quantify its discriminative performance, which was operated with a 1000-times bootstrap process. The time-dependent receiver operator characteristic (ROC) curve was applied to evaluate the discriminative ability to predict survival outcomes at 3, 5, and 10 years. Calibration curves were used to assess the accuracy of the prognostic model when predicting short-, mid-, and long-term OS probabilities. Clinical utility was assessed based on decision curve analysis (DCA).

The established nomogram was validated in an independent CGGA cohort by evaluating its reliability; whereas ROC, calibration, and DCA curves were calculated to assess its discriminative ability, robustness, and clinical value, respectively.

### Pathway Enrichment and Correspondence Between ERGs and eRNAs

Each ERG was subjected to gene set enrichment analysis (GSEA) to identify related Kyoto Encyclopaedia of Genes and Genomes pathways and biological processes. The required gene-rank lists were generated based on Pearson correlations between each ERG and all other genes in the training cohort (18, 19). The selection criteria for enriched pathways were defined as a false discovery rate < 0.05 and a simultaneous absolute value of the enrichment score > 0.5.

TCGA and eRic data were used to analyze the correspondence between eRNAs and ERGs, as well as between different seRNAs that regulated the same ERGs and were located in neighbouring chromosomal sequences. ERGs were separated into various groups based on whether they were regulated by specific eRNAs or seRNAs.

### Tumor Immune Landscape Comparison in Different Risk Groups

Different signature groups were compared in terms of immune-related profile mutations to reveal possible immune mechanisms involved in the progression of gliomas. The tumor microenvironment (TME) and immune responses modulate cancer progression through interactions with tumors (20). Therefore, to determine the relative proportion of tumor and immune cells in the TME, immune scores and tumor purity were estimated for each patient using the *estimate* package in R. The connection would be found out between the established model and immune infiltration profile, based on the model’s linear prediction value and TME profile. In addition, the TIMER dataset was used to explore the Spearman correlation between ERG risk score and immune infiltration levels of six main immune cell types, i.e. B cells, CD4^+^ T cells, CD8^+^ T cells, neutrophils, macrophages, and dendritic cells. To obtain a therapeutic reference, the tumor mutation burden (TMB) and key immune checkpoint biomarkers, such as programmed cell death 1 ligand 1 (PD-L1) and cytotoxic T-lymphocyte–associated protein 4 (CTLA-4), were compared among low-, mid-, and high-risk groups.

### Evaluation of the ERGs Risk Signature

Some important biomarkers were not incorporated into the prognostic model, because there not could be provided by the CGGA cohort and the established model would not be externally validated, including epidermal growth factor receptor (EGFR) amplification, TERTp mutation status, and the combination of whole chromosome 7 gain and whole chromosome 10 loss (chr 7+/10−). More importantly, they were all significant biomarkers to affect the survival outcome and often resulted in serious outcomes for LGG patients who had been referred to as ‘molecular grade IV patients’ (17). Hence, to make full use of their information, they were all used as reference biomarkers to evaluate the predictive value of the ERGs risk signature.

To evaluate the prognostic value of the ERG risk signature about existing biomarkers, the integrated discrimination improvement (IDI), continuous net reclassification index (cNRI), and incremental area under the curve (iAUC) were calculated. These indicators of the ERG risk score represented the incremental difference between Model 1 and Model 2. Model 1 included prognostic indicators of age, sex, radiotherapy, isocitrate dehydrogenase (IDH) status, 1p19q codeletion, MGMT methylation, TERT gene expression, EGFR amplification, and chr 7+/10-. The corresponding 95% confidence intervals (CIs) were acquired using the bootstrap procedure.

### Genomic Mutation of the ERGs

Genomic mutation was another important driver factor to affect the abnormal expression of target genes. To verify the specificity of the driver factor of eRNA, we used the *TCGAWorkflow* R package to analyze the genomic mutation for all LGG patients, including the single nucleotide variants and somatic copy-number alteration.

### Vitro Experiment Validation to Specific eRNA-Regulated Gene

#### Cell Culture and Transfection

U251 and Hs 683 were types of glioma cells, commercially obtained from the Cell Bank of the Chinese Academy of Sciences (Beijing, China). U251 and Hs 683 were maintained in DMEM-6429 (Sigma, MO, USA) containing 5% (10% for Hs 683) fetal bovine serum (FBS, HyClone, Logan, UT, USA) at 37°C in a 5% CO2 atmosphere. Small interfering RNAs targeting USP28 (si-USP28-1: 5’-GAUUAUAGUUUGUUCCGAATT-3’, 5’-UUCGGAACAAACUAUAAUCTT-3’ and si-USP28-2: 5’-UUGGUUUAGUGCUGUUAUUCTT-3’, 5’-AAUAACAGCACUAAACCAATT-3’), negative controls (si-USP28-NC) were purchased from Nantong Biomics Biotechnologies company. Lipofectamine 2000 (Thermo Fisher Scientific, Inc) was used for transfection according to the manufacturer’s instructions. The cells were harvested 48 hours after transfection.

#### Protein Extraction and Western Blot Analysis

Total proteins were extracted from the cells with RIPA buffer and quantified by a BCA kit (Beyotime Biotechnology). About 30 μg of extracted proteins were separated by SDS-PAGE and then transferred onto PVDF membranes (Merck Millipore). After being soaked with 5% non-fat milk for 2h at 25°C and incubated with USP28 and GAPDH, the PVDF membranes were eventually incubated with a secondary antibody (Cell Signaling Technology).

#### Cell Viability and Colony Formation Assays

EdU (5-Ethynyl-2’-deoxyuridine) staining was performed to evaluate cell viability. The transfected cells were seeded into 96-well plates, and the proliferation was examined using a commercial EdU Kit (UE, China) according to the manufacturer’s protocol. Images were obtained using a fluorescence microscope (Leika, Germany) and analyzed with Image J. To detect the clonogenic capacity, a colony formation assay was carried out. The transfected cells were seeded into 35 mm culture dishes and cultivated with DMEM-6429 containing 5% (10% for Hs 683) FBS. Cell colonies were fixed with paraformaldehyde and stained with 0.1% crystal violet (Beyotime) for 20 minutes, and then colony counting was determined by microscope.

#### Transwell Assay

The transfected cells were suspended in a serum-free medium and plated into the transwell chambers with a pore size of 8 µm. Cell invasion was evaluated performing the Chamber matrigel invasion 24-well units (Costar). The assays were performed according to the manufacturer’s instructions. Briefly, cells from each group were suspended in a serum-free medium and were seeded into the upper chamber. The lower chamber was filled with medium containing 10% FBS. After incubation for 24 hours, the migrated/invaded cells in the lower chamber (below the filter surface) were fixed in 4% paraformaldehyde, stained with crystal violet solution and counted under a microscope.

#### Evaluation of Cell Apoptosis

Cell apoptosis was determined by Annexin V-FITC/PI Apoptosis Detection Kit (BD Pharmingen, USA), and quantified by flow cytometry. Briefly, after inducing apoptosis, 1×105 cells of each group were harvested and resuspended in 300 μL binding buffer containing 5 μL Annexin V-FITC for 30 min at 4°C in the dark, followed by further incubation with 5 μL PI for 5 min. Samples were then analyzed with a FACSCanto II equipped with FACSDiva software (BD Bioscience). Live cells were identified as Annexin V-FITC-/PI- (lower left quadrant), apoptotic cells as Annexin V-FITC+/PI+ (upper right quadrant).

#### Verification of USP28 Expression in Glioma

To verify the expression of USP28 in glioma, evidence was provided in two ways, including population cohort gene expression detection and immunohistochemistry (IHC) analysis. The former was obtained from the Gene Expression Omnibus (https://www.ncbi.nlm.nih.gov/geo/; GEO ID: GSE4290). The latter was obtained from The Human Protein Atlas (https://www.proteinatlas.org/), which contained IHC data using a tissue microarray-based analysis on the different normal tissue types, and proteome analysis of the major cancer types. Staining intensity, quantity, location, and patient’s information in patients with the respective cancer types were available online.

### Statistical Analysis

All statistical analyses were performed using R software (www.r-project.org; version 4.0.3) and were two-sided, including the Wilcoxon test, Kruskal-Wallis test, correlation test, chi-square test, and proportional hazards assumption test. The proportional hazards assumption test was conducted the by Schoenfeld method using the *survival* R package. Results with a *p-value* < 0.05 were considered statistically significant.

## Results

### Survival-Related eRNAs and Prognostic ERGs

Gene expression and clinical data, eRNA expression data, immune infiltration data and copy number variation data of 530 LGG patients were collected from the TCGA, eRic, TIMER and cBioPortal datasets. Patients whose missing clinical and survival information was MCAR (Little’s test, *p* > 0.05) were directly filtered out. Eventually, 428 patients satisfied the inclusion criteria and were used for further analyses. In the CGGA cohort, 399 LGG patients were selected after the batch effect (325 and 693 patients in two batches) was corrected and MCAR inclusion criteria were satisfied (*p* > 0.05).

From the first dataset, 1214 LGG eRNAs were obtained and subjected to Kaplan-Meier and Cox analyses, which identified 346 prognosis-related eRNAs (**Table S1**). In the same dataset, of the 623 target ERGs, 289 were selected as candidate prognostic ERGs by Kaplan-Meier analysis. Filtering through the LASSO Cox model identified 14 prognostic ERGs (**Table S2** and **Figure S1**), of which 13 were retained because they displayed a strong correlation between mRNAs and their regulating eRNAs (**Table 1**, *r_s_* > 0.3, *p* < 0.05). The specific survival analysis for each gene showed that they were all closely correlated to the survival outcomes (**Figure S2**; all *p* < 0.05).

**Table 1:** The relationship between eRNA and ERG.

Gene type	Gene	eRNA	enhancer site	eRNA tissue	*r_s_*
seRNA-regulated	CDKN2C	1:51006793-51012793	1 (51006793-51012793)	multi-tumors	0.82^*^
		1:51007228-51013228	1 (51007228-51013228)	multi-tumors	0.83^*^
seRNA-regulated	GNG12	ENSR00000008301	1 (68090933-68096933)	multi-tumors	0.70^*^
		ENSR00000252382	1 (68090200-68096200)	multi-tumors	0.68^*^
seRNA-regulated	RYR3	ENSR00000074563	15 (32464000-32470000)	multi-tumors	0.46^*^
		ENSR00000074564	15 (32466200-32472200)	multi-tumors	0.44^*^
		ENSR00000074565	15 (32467300-32473300)	multi-tumors	0.43^*^
seRNA-regulated	SEMA4G	ENSR00000032650	10 (101831600-101837600)	LGGs	0.61^*^
		ENSR00000032651	10 (101833177-101839177)	LGGs	0.60^*^
		ENSR00000261154	10 (101833100-101839100)	LGGs	0.60^*^
seRNA-regulated	ZSCAN20	ENSR00000004535	1 (33498973-33504973)	multi-tumors	0.62^*^
		ENSR00000251119	1 (33497800-33503800)	multi-tumors	0.72^*^
		ENSR00000251120	1 (33499000-33505000)	multi-tumors	0.62^*^
specific eRNA-regulated	NRG3	ENSR00000030804	10 (81863945-81869945	LGGs	0.63^*^
specific eRNA-regulated	PPM1L	ENSR00000161287	3 (161260322-161266322)	LGGs	0.51^*^
specific eRNA-regulated	RGR	ENSR00000260547	10 (84261800-84267800)	LGGs	0.94^*^
specific eRNA-regulated	TBPL1	ENSR00000203159	6 (134106735-134112735)	LGGs	0.57^*^
specific eRNA-regulated	USP28	ENSR00000265929	11 (113246600-113252600)	LGGs	0.36^*^
other	ARL3	ENSR00000032851	10 (103194700-103200700)	multi-tumors	0.50^*^
other	CUEDC2	ENSR00000032851	1 (51006793-51012793)	multi-tumors	0.39^*^
other	EIF2AK4	ENSR00000075512	15 (40395501-40401501)	multi-tumors	0.40^*^

seRNA-regulated, super-enhancer eRNAs regulated gene; specific eRNA-regulated, specific eRNAs regulated gene; r_s_, Spearman’s correlation coefficient; *, the statistically significant result (p < 0.05).

**Figure 2:**
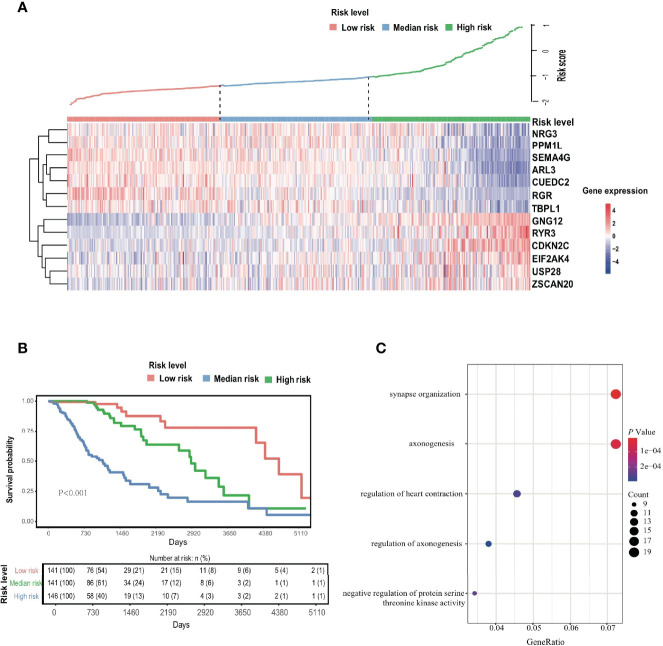
EDGs signature. **(A)** The Heatmap of 13 screened EDGs expression and distribution of corresponding risk scores among low-, mid- and high-risk subgroups in the TCGA cohort. **(B)** The Kaplan-Meier analysis for the risk signature. **(C)** The GO analysis for 289 candidates EDGs.

To evaluate the comprehensive prognostic effect of the above 13 ERGs in LGG, the risk score was calculated for each individual using the inner product of gene expression level and relative coefficient in the LASSO regression the risk score was calculated by the following formula: (Risk score = -0.20 * ARL3 + 0.10 * CDKN2C - 0.10 * CUEDC2 + 0.14 * EIF2AK4 + 0.10 * GNG12 – 0.10 * NRG3 – 0.15 * PPM1L – 0.13 * RGR + 0.30 * RYR3 – 0.45 * SEMA4G – 0.01 * TBPL1 + 0.26 * USP28 + 0.12 * ZSCAN20). Based on the risk score, 428 LGG patients were classified into low-risk (141 members), mid-risk (141 members), and high-risk (146 members) groups. Gene expression profiles for LGG patients with different risk signatures are reported as a heatmap (**Figure 2A**). Kaplan-Meier analysis among the three groups revealed that a higher risk signature corresponded to a lower survival probability (*p* < 0.05; **Figure 2B**).

At the same time, GO enrichment analysis on 289 candidate genes identified five GO terms (adjusted *p* < 0.05; **Figure 2C** and **Table S3**), whose functions were related mostly to the regulation of synapses and axonogenesis.

### Prognostic Tool, Internal Validation, and External Validation

**Figure 3:**
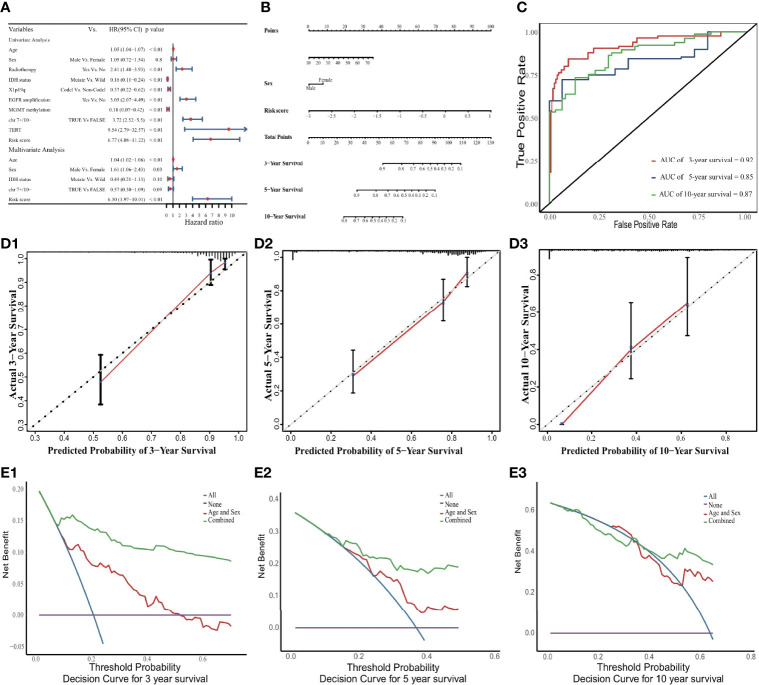
Nomogram to predict 3-, 5, and 10-year OS. **(A)** Univariate and multivariate Cox analyses. The limitation of CI is defined by the arrow symbols. **(B)** Nomogram to predict the 3-, 5- and 10-year OS for LGG patients. **(C)** Time-dependent ROC curves for 3-, 5- and 10-year OS prediction. **(D)** Calibration curves of 3-, 5- and 10-year OS. The black diagonal lines represent the ideal performance in predicting OS, and the red lines represent the actual performance. **(E)** The DCA curves.

A comparison of demographic indicators and clinical features including age, sex, radiotherapy status, IDH status, 1p19q codeletion, MGMT methylation, TERT gene expression and risk signature, revealed that only risk score, age, and sex were significant factors in the prognostic model (**Figures 3A, B** **)**. The relevant clinicopathologic and prognostic model information of all patients had been described in **Table S4**. It could find that the MGMT methylation level was closely related to the IDH status (*p* < 0.001; **Figure S3**). A model that included only age, sex and the ERG risk score as significant predictive prognostic indicators achieved a C-index of 0.86 (95% CI: 0.83-0.90), which represented a good discriminative ability. Its proportional hazards assumption test was not statistically significant (*p* = 0.88; **Figure S4** and **Table S5**). Similarly, the survival probabilities predicted by the prognostic model were significantly higher in the low-risk group than in the high-risk group according to Kaplan-Meier analysis (*p* < 0.001; **Figure S5**).

In the internal validation cohort, the discriminative ability of the prognostic model was demonstrated using time-dependent ROC curves (**Figure 3C**). Calibration curves displayed promising capability when predicting prognostic outcomes at 3, 5, and 10 years (**Figure 3D**). DCA revealed a clear advantage of the nomogram over the combination of age and sex (**Figure 3E**).

**Figure 4:**
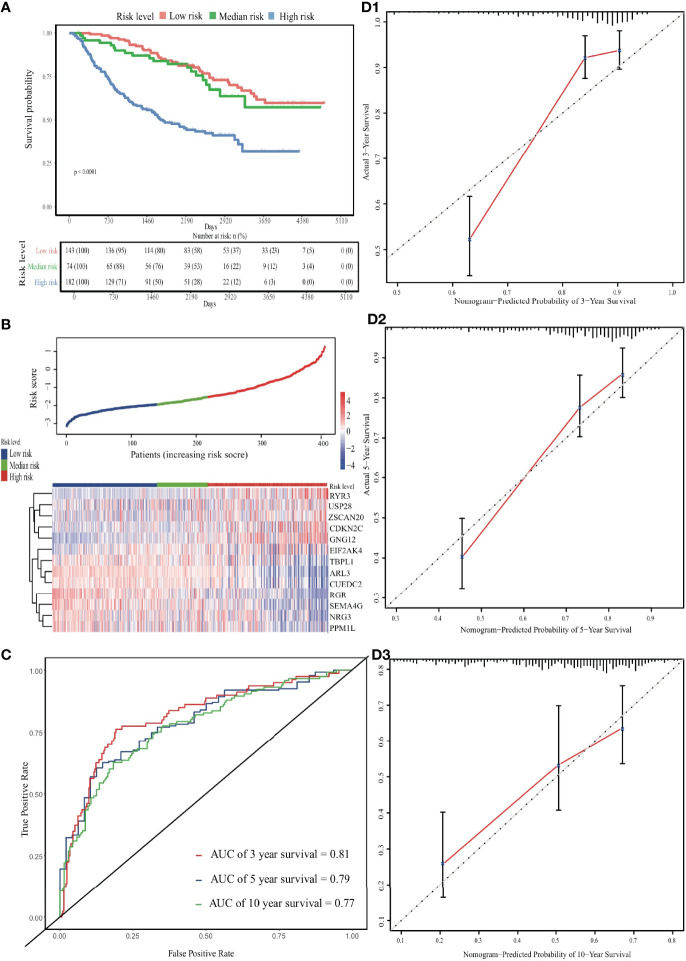
Validation of the EDGs nomogram. **(A)** The Kaplan-Meier analysis of the risk scores. **(B)** The heatmap and distribution of EDGs expression profile among three subgroups in CGGA cohort. **(C)** Time-dependent ROC curves for 3-, 5- and 10-year OS prediction. **(D)** The calibration curves for the OS nomogram in the CGGA cohort. The black diagonal lines represent the ideal performance in predicting OS, and the red lines represent the actual performance.

In the external validation cohort, the predicted survival probabilities differed significantly between the low-risk and high-risk groups (*p* < 0.001; **Figure S6**). Among the three risk groups, OS and gene expression profile differences related to the ERG signature are shown in **Figures 4A, B**. Although lower than in the internal validation cohort, the predictive ability remained at a relatively satisfactory level. The AUC for time-dependent ROC curves was 0.81, 0.79 and 0.77 at 3, 5, and 10 years, respectively (**Figure 4C**). Calibration curves performed well in the independent validation cohort (**Figure 4D**).

### Pathways, Potential Mechanisms, and Regulatory Interactions Between eRNAs and ERGs

**Figure 5:**
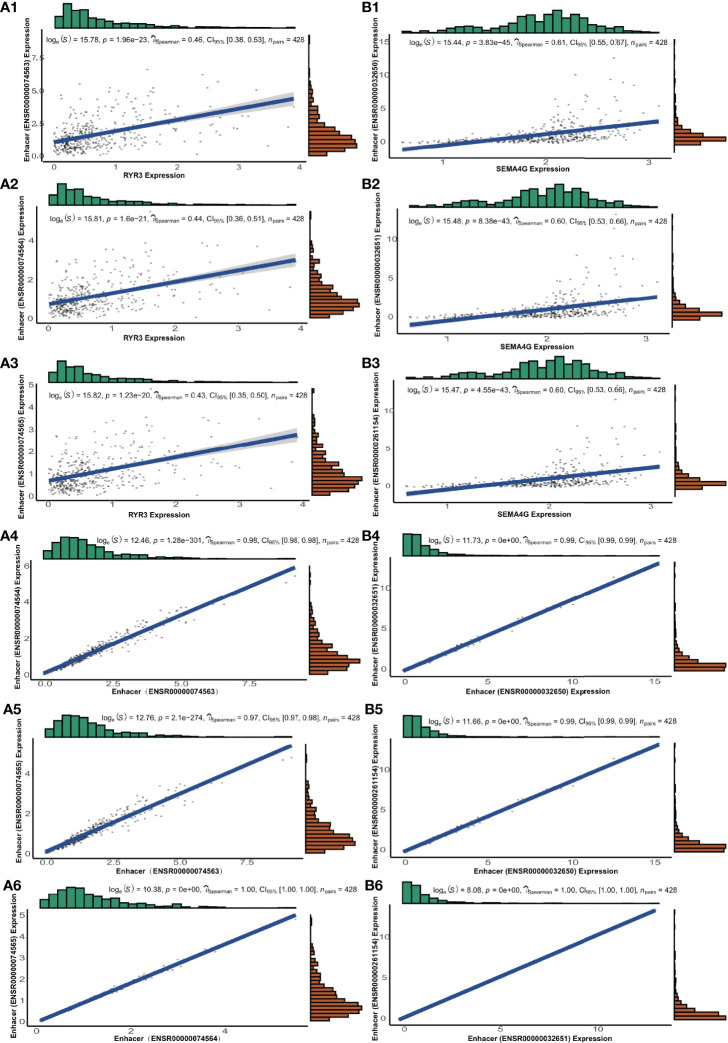
The genes of RYR3 and SEMA4G and their regulative super-enhancers. **(A1-A3)** The correlation between the expression and its regulative eRNAs of RYR3. **(A4-A6)** The correlation among regulative eRNAs of RYR3. **(B1-B3)** The correlation between the expression and its regulative eRNAs of SEMA4G. **(B4-B6)** The correlation among regulative eRNAs of SEMA4G.

For each pair of eRNAs and their ERGs, significant positive correlations were found (*r_s_* > 0.3, *p* < 0.05) in the TCGA cohort. Notably, five target genes, including CDKN2C, GNG12, RYR3, SEMA4G, and ZSCAN20, were regulated by seRNAs. Besides localizing to adjacent sites, in the case of SEMA4G and RYR3, the components of these super-enhancers associated strongly also with their neighbouring partners (*r_s_* > 0.9; *p* < 0.05; **Figures 5A, B** **)**. In addition, five other genes, including TBPL1, USP28, NRG3, PPM1L, and RGR, were regulated by eRNAs expressed only in LGG tumor tissue; whereas SEMA4G was regulated by LGG-specific seRNAs (**Table 1**).

**Figure 6:**
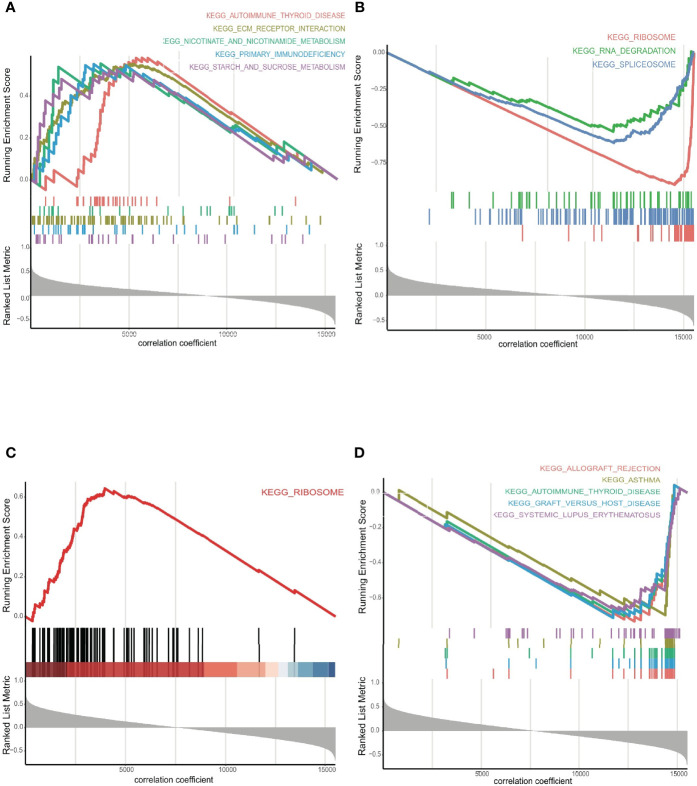
**(A)** The GSEA for RYR3 in positive, and **(B)** negative groups, respectively. **(C)** The GSEA for SEMA4G in positive, and **(D)** negative groups, respectively.

GSEA revealed that candidate ERGs were involved predominantly in immune-related and cancer-related pathways, such as ‘primary immunodeficiency’, ‘allograft rejection’, ‘autoimmune thyroid disease’, ‘ribosome’, ‘spliceosome’, and ‘deregulation of the cell cycle’ (**Tables S6** **,** **S7**). Interestingly, RYR3 and SEMA4G were the most important harmful and protective genes, as indicated by their large regression coefficients (0.30 and -0.45, respectively) in the LASSO-Cox model. Both were also regulated by seRNAs consisting of three enhancers. For RYR3, the top five signaling pathways in the positive correlative group were ‘autoimmune thyroid disease’, ‘ECM receptor interaction’, ‘nicotinate and nicotinamide metabolism’, ‘primary immunodeficiency’, and ‘starch and sucrose metabolism’ (**Figure 6A**); whereas the most significant signalling pathways in the negative correlative group were ‘ribosome’, ‘RNA degradation’, and ‘spliceosome’ (**Figure 6B**). For SEMA4G, the most important pathways enriched in the positive and negative correlative groups were ‘ribosome’, ‘allograft rejection’, ‘asthma’, ‘autoimmune thyroid disease’, ‘graft versus host disease’, and ‘systemic lupus erythematosus’ (**Figures 6C, D** **)**.

### Tumor Immune Microenvironment

**Figure 7:**
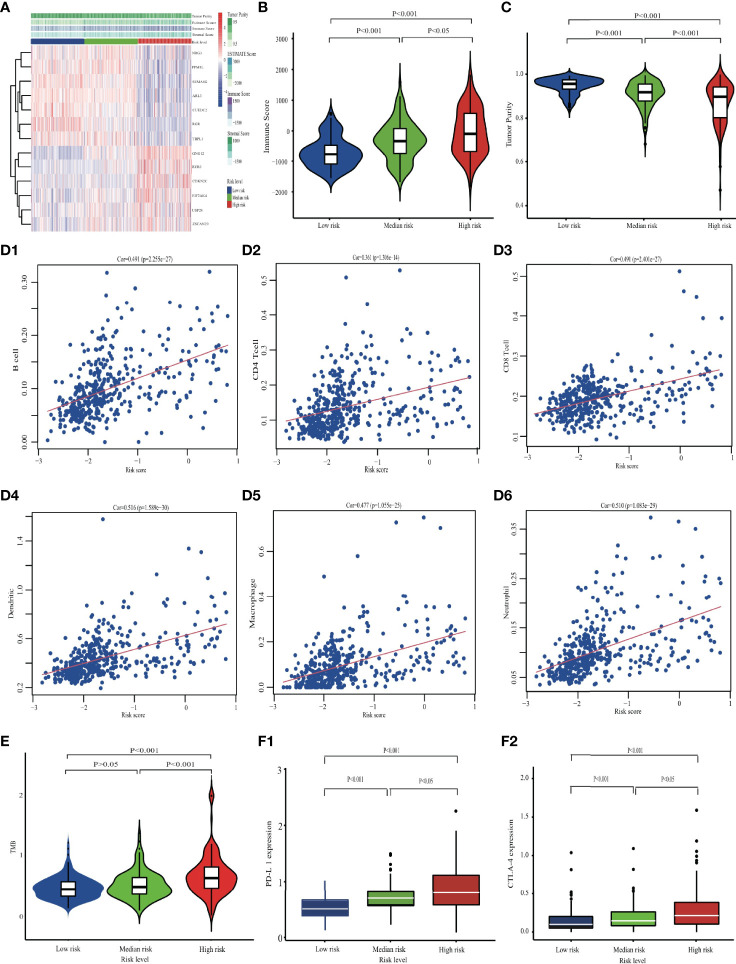
Comparing the immune characteristics among different risk groups in the TCGA cohort. **(A)** The overall immune microenvironment landscape. **(B)** The comparison of Immune score, and **(C)** tumor purity. **(D)** The correlation between six immune cells and EDGs signature. **(E)** Differences in TMB among low-, mid- and high-risk groups. **(F)** The differences in checkpoint expression.

A comparison of the three groups revealed significantly higher overall immune infiltration with increasing risk level (*p* < 0.001; **Figures 7A, B**), but concomitant decreased tumor purity in the TME (*p* < 0.001; **Figure 7C**). The prognostic model was statistically related to TME profiles, including immune score, stromal score, estimated score, and tumor purity, which maintained a similar tendency with ERG risk signature (*p* < 0.001; **Figure S7**). Six main immune cell types were positively associated with ERG risk (*p* < 0.001; **Figure 7D**). The TMB was higher in the high-risk group than in low- or mid-risk groups (*p* < 0.001; **Figure 7E**). Finally, a comparison of gene expression levels showed that immune checkpoint genes PD-L1 and CTLA-4 were upregulated at increasing risk signatures (*p* < 0.001; **Figure 7F**).

### Prognostic Value of the ERG Risk Signature

Although all of the genetic biomarkers were significant in the univariate Cox model, only the chr 7+/10- and the ERGs risk signature were important prognostic factors in the multivariate Cox model, which indicated that the importance of the ERGs risk signature was reliable and it could provide crucial information when used to predict the OS for LGGs patients (**Table S8**).

Incremental IDI, cNRI, and iAUC values confirmed the prognostic reliability of the ERG risk signature (**Table 2**). In particular, IDI was improved by > 10%, which meant that the ERG risk signature could significantly increase the predictive accuracy of LGG patient survival outcomes compared with several genetic biomarkers currently in clinical application.

**Table 2:** The predictive evaluation index for ERGs risk signature.

Timepoint	IDI (95% CI)	cNRI (95% CI)	cNRI^+^ 95% CI	cNRI^-^ (95% CI)	iAUC (95% CI)
3 year	0.11 (0.04 - 0.20)^*^	0.47 (0.18 – 0.81)^*^	0.34 (0.12 - 0.55)^*^	0.13 (0.01 - 0.33)^*^	0.06 (0.01 - 0.12)^*^
5 year	0.10 (0.03 - 0.18)^*^	0.49 (0.10 - 0.77)^*^	0.18 (-0.02 - 0.38)	0.31 (0.04 - 0.48)^*^	0.04 (-0.01 - 0.12)
10 year	0.22 (-0.04 - 0.58)	0.66 (0.07 - 1.19) ^*^	0.26 (0.04 - 0.52)^*^	0.40 (-0.07 - 0.78)	0.12 (-0.01 - 0.30)

IDI, integrated discrimination improvement; cNRI, continuous net reclassification index with cutoff 0.05; iAUC, incremental AUC; *: the statistically significant result. IDI was used to evaluate the increment of predictive accuracy; cNRI and iAUCs were used to evaluate the increment of the discriminative accuracy when the ERGs risk score was additionally included in the prognostic model. Improvement in risk prediction was tested with IDI, cNRI and iAUC by adding ERGs risk score on two multivariable models: model 1 included age, sex, radiotherapy, IDH status, 1p19q codeletion, MGMT methylation, EGFR amplification, TERT gene and chr 7+/10-; model 2 was model 1 plus the ERGs risk score.

### The Genomic Mutation of the ERGs

Although the genomic mutation status of all LGG patients was analyzed, the mutation frequencies (< 1%) were too low to consider the genomic mutation as the driving factor to result in abnormal ERGs expression (**Figure S8**). Most of the LGG patients had nothing genomic mutation in single nucleotide variants and somatic copy-number alteration for the ERGs.

### Knockdown of USP28 Inhibits U251 and Hs 683 Cell Proliferation, Invasion and Apoptosis *In Vitro*

**Figure 8:**
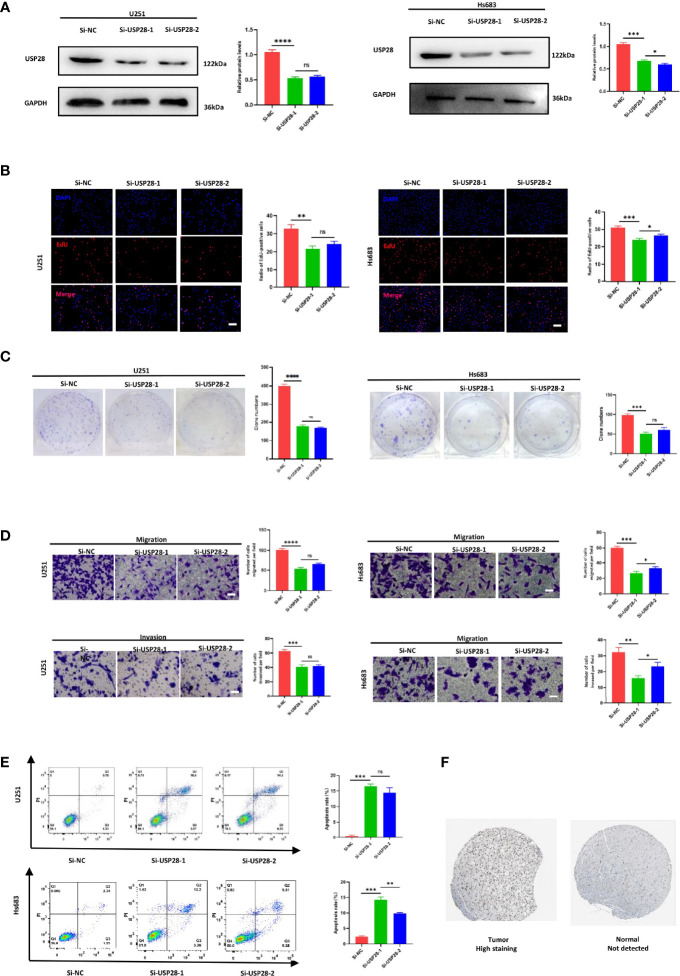
Knockdown of USP28 suppressed the proliferation, migration, invasion and increased cell apoptosis rate of U251 and Hs 683 cells *in vitro*. **(A)** Western blot analysis to examine the efficiency of the USP28 knockdown. **(B)** Proliferation ability in USP28 knockdown U251 and Hs 683 cells by EdU staining. **(C)** Colony-forming abilities in USP28 knockdown U251 and Hs 683 cells by clonogenic assays. **(D)** Transwell assays to detect the migration and invasive capacities in USP28 knockdown U251 and Hs 683 cells. **(E)** Flow cytometry to analyze the apoptosis of U251 and Hs 683 cells. **(F)** USP28 expression in glioma tissues. Magnification, × 200 **(B, D)**. Scale bar, 100 μm **(B, D)**. *, **, ***, **** and ns mean p < 0.05, p < 0.01, p < 0.001, p < 0.0001 and insignificance in statistics, respectively. Data are shown as mean ± SD of at least three independent experiments.

Two siRNAs (si-USP28-1 and si-USP28-2) targeting USP28 were transfected into the U251 and Hs 683 cells. according to the western blot analysis, both of the two selected siRNAs could significantly decrease USP28 expression as shown in **Figure 8A**. Next, EdU staining and colony formation assays were performed to assess the cell proliferation. The results indicated that compared with siRNA of negative control (si-NC), the silence of USP28 significantly suppressed cell growth (**Figure 8B**), and the formation of tumor cell colonies (**Figure 8C**).

We further explored the potential impact of USP28 on migration and invasion by transwell assays. U251 and Hs 683 cells transfected either with si-USP28-1 and si-USP28-2 presented a dramatically inhibited migration and invasion ability (**Figure 8D**). Interestingly, the apoptosis rates were both higher in the USP28 silence group than the negative control (**Figure 8E**). Those results suggested a critical role of USP28 in the cell proliferation and aggressiveness of glioma cells.

### USP28 Expression in Glioma

The GEO cohort dataset included 180 samples, including 23 normal samples, 76 LGG samples, and 81 GBM samples. The expression levels of USP28 gradually and statistically increased with the severity of the disease (*p* < 0.05; **Figure S9**). Besides, the IHC staining showed that USP28 was not expressed and translated in normal cerebral cortex tissues, while its expression in LGG tissues was observed with higher levels (**Figure 8F**). Taken together, these results indicated that USP28 was highly expressed and translated in LGG tissues, compared with normal cerebral cortex tissues.

## Discussion

LGGs are some of the most common tumors in the central nervous system. However, the heterogeneity and complexity of these tumors have delayed the development of specific and effective predictive biomarkers for LGGs (21, 22). An effective prognostic model based on specific biomarkers could accurately forecast survival outcomes, allowing efficient management of patients with LGGs. Compared with other genetic biomarkers, eRNAs have the advantage of being highly tissue-specific. Therefore, genes targeted by eRNAs were hypothesized to be good candidates for the prognosis of LGGs (9, 23). Here, we constructed an ERG risk score and further combined it with age and sex to develop a prognostic model for individual prediction of LGG outcomes. The performance of the prognostic model was verified in training and external validation cohorts, which confirmed its robustness and reliability for short-, mid-, and long-term survival prediction. Therefore, the model could help clinicians make more accurate assessments, prescribe niche targeting therapies, and propose more rational post-discharge management. The prognostic value of the risk signature was confirmed by IDI, NRI, and iAUC indicators, and compared with clinically applied biomarkers, such as IDH status, 1p19q codeletion, EGFR amplification, TERT mRNA expression level, and chr 7+/10- status. In the multivariate model, the IDH status had minor importance to survival outcomes compared with age, sex, and the EGR risk score. But the MGMT methylation level was not directly included in the prognostic model, which may be attributed to the close relationship between IDH status and MGMT methylation level. Although the somatic mutation had been thought as an important driver factor to affect the abnormal gene expression and survival outcomes (24). But we had found that the ERGs we identified might not be affected by the genomic mutations, which reinforced the belief that the eRNAs were the key driving factors to affect the abnormal expression of ERGs. Far from being limited to gliomas, eRNAs and their ERGs have great potential for the diagnosis of various other tumors, therapeutic target identification, and prognostic evaluation.

Malfunctioning of the enhancer or super-enhancers, which is strongly associated with aberrant eRNA expression, is now considered a key driving cause of tumor onset and progression (9, 25). As eRNA levels are significantly positively related to target gene expression levels, prognoses could be improved by regulating eRNAs, particularly those specific to LGGs. For example, Spearman’s correlation coefficient > 0.9 was found between the RGR target gene and its LGG-specific eRNA regulator (ENSR00000260547). Hence, ERGs or eRNAs would be promising prognostic and immunotherapeutic/therapeutic targets because of their elevated specific expression in tumors.

In the prognostic model based on thirteen ERGs, five were identified as seRNA-regulated genes, of which three were immune-related, five ERGs were regulated by tissue-specific eRNAs including two immune-related ones, and two more immune-related genes were identified among the remaining three ERGs. The most important immune-related gene in the LASSO-Cox model was SEMA4G, which was regulated by specific seRNAs (22). Evidence indicated that the survival of LGG patients was strongly affected by changes to the immune function in tumor tissues, prompting us to further investigate the relationship between tumor immune features and the ERG signature. The risk group with the highest ERG score was found to have low tumor purity but elevated immune cell infiltration. TIMER data revealed a significant positive association between the ERG score and the infiltration levels of six common immune cell types, with heavier immune infiltration leading to worse survival outcomes. Tumor purity and immune infiltration are vital parameters in tumor prognosis, and low tumor purity is closely associated with poor prognosis in glioma (26–28). If glioma cells have lower proliferative and invasive clinical properties, they tend to form a stable solid tumor with fewer noncancerous infiltrating cells such as immune cells. Generally, the occurrence of an overly intensive immune response in tumor tissues, in which macrophages and neutrophils can recruit immune cells to establish their protective shields, leads to a poor prognosis in glioma (28, 29). In the TME, the aggregation of macrophages, including M1 and M2 phenotypes, generally contributes to tumor growth and invasion (30). Furthermore, the aggregation of neutrophils points to tumor grade progression, treatment resistance, and shorter survival, which occur as a result of local immunosuppression and inhibition of beneficial natural killer cells and CD8^+^ T cells (31, 32). The significant correlation between ERGs and the immune microenvironment indicated that OS could be prolonged by controlling enhancer activity and eRNA expression, especially that of eRNAs responsible for regulating immune-related genes.

To determine the relationship between immune features and ERG signature, we assessed the immunotherapeutic significance of the latter. Although advances in immunotherapy have greatly facilitated the treatment of malignant tumors, immune resistance remains a serious problem in clinical practice. As a result, it is not always clear, which patients may benefit from it (33). Tumor immune escape allows tumor cells to adapt to immune resistance (23). The PD-L1 checkpoint is involved in the negative regulation of T-cell activation, which can mitigate the inflammatory response, maintain immune homeostasis, and promote immunosuppression (34). Checkpoint inhibitors are a ground-breaking tool in cancer immunotherapy and have achieved satisfactory therapeutic effects against malignant tumors. The levels of checkpoint proteins and the TMB represent effective biomarkers for predicting the effects of anti-checkpoint immunotherapy (20). Elevated PD-L1 expression in tumor cells is an established biomarker associated with improved clinical response to checkpoint blockade. Similarly, patients with a high TMB tend to display a better response to immunotherapy than those with a lower TMB (35). Our results point to promising immunotherapeutic effects in high-risk LGG patients due to their elevated checkpoint biomarker levels and TMB.

Confirming the significance of immune landscape analysis in this study, GSEA revealed the key role of immune-related pathways in LGG patients. Several pathways were involved in tumor prognosis, providing novel insights on the molecular mechanisms required for predicting LGG (36–38). ‘Spliceosome’, ‘ribosome’, and ‘deregulation of the cell cycle’ were the main functionally related pathways involving ERGs. The formation of non-functional spliceosomes causes defects in RNA processing, which could be deleterious to cells and affect oncogenic factors in multiple types of tumors. Indeed, aberrant splicing has been documented in glioblastoma (39). The ribosome pathway is associated with ribosome biogenesis and protein synthesis, whose increased activity could promote mRNA translation and cell growth (40, 41). Deregulation of the cell cycle underlines another important mechanism for tumor pathogenesis and progression, as it can lead to malignant cell proliferation (42). Overall, a perturbed ERG expression could influence spliceosome formation and ribosome activity, thereby affecting the cell cycle, glioma cell proliferation and, ultimately, OS.

USP28 was expressed in many cancers and had different biological mechanisms. A large number of studies had shown that targeting USP28 would have potential therapeutic effects on a variety of cancers, including non-small cell lung cancer, breast cancer, colon cancer, glioma and bladder cancer (43). According to a series of bioinformatics and experiments, we found that silencing USP28 in U251 and Hs 683 cells, significantly decreased cell viability, clone formation, migration and invasion ability, and induced cell apoptosis. The population data and IHC results all indicated that the USP28 were highly expressed and translated in glioma tissues, compared with normal tissues.

The present study has some limitations, which should be addressed by future investigations. First, because the prognostic model could be built only in a cross-sectional study, it is necessary to acquire more reliable prospective evidence before using it in clinical practice. Second, the chromosomal location of certain enhancers, and the direct regulatory relationships between eRNAs and their ERGs should be verified. Third, multi-omics data on DNA methylation, microRNA, and long non-coding RNA should be taken into consideration to better understand the regulation of gene expression. Fourth, only a specific eRNA-regulated gene was robustly validated in our study, and the rest of them should be done the similar validation in future studies. Finally, other important biomarkers described in the third edition of the Consortium to Inform Molecular and Practical Approaches to CNS Tumor Taxonomy, such as chr 7+/10-, and TERTp mutation, should be evaluated (44).

In conclusion, we identified thirteen ERGs associated with OS in patients with LGGs and blended them into a comprehensive ERG signature. Then, the ERG features were combined with age to develop a nomogram for predicting individual OS, which displayed elevated sensitivity and specificity, and may contribute to more rational clinical management. To some extent, the good performance of the established ERG prognostic model could be attributed to the high tissue specificity of eRNAs. Additionally, this study has identified ERGs whose regulatory eRNAs or enhancers, or the ERGs themselves, could serve as prospective diagnostic and therapeutic biomarkers. This is particularly true for LGG-specific biomarkers, such as SEMA4G and its putative eRNA and enhancer. Moreover, owing to their high tissue specificity, eRNAs could help predict OS also for other tumors.

## Data Availability Statement

The original contributions presented in the study are included in the article/**Supplementary Material**. Further inquiries can be directed to the corresponding authors.

## Author Contributions

ML and QJZ designed the study. WT, XH, and GY analyzed the data and prepared the manuscript. KC and GY finished the biological validation experiment. All authors were substantially involved in the research, acquisition of data, analysis, manuscript preparation, and read and approved the final manuscript.

## Funding

This work was supported by funds from the National Science and Technology Major Project of the Ministry of Science and Technology of China (grant number2016ZX08011005), the National Natural Science Foundation of China (grant number 82073666) and the National Science Foundation for Young Scientists of China (grant number 81502889).

## Conflict of Interest

The authors declare that the research was conducted in the absence of any commercial or financial relationships that could be construed as a potential conflict of interest.

## Publisher’s Note

All claims expressed in this article are solely those of the authors and do not necessarily represent those of their affiliated organizations, or those of the publisher, the editors and the reviewers. Any product that may be evaluated in this article, or claim that may be made by its manufacturer, is not guaranteed or endorsed by the publisher.

## Supplementary Material

Supplementary Figure 1 The resulting graph of the LASSO cox model. **(A)** The selection of lambda penalty parameter for the Lasso-Cox model. **(B)** The coefficient change curve of variables.

Supplementary Figure 2 The Kaplan-Meier curve for each ERG.

Supplementary Figure 3 The correlation between MGMT methylation and IDH status.

Supplementary Figure 4 The PH test figure for the prognostic model.

Supplementary Figure 5 The Kaplan-Meier curve for the nomogram in the TCGA cohort.

Supplementary Figure 6 The Kaplan-Meier curve for the nomogram in the CGGA cohort.

Supplementary Figure 7 The correlation between prognostic model and immune infiltration profiles. **(A)** correlation analysis for the immune score; **(B)** for the stromal score; **(C)** for estimate score; **(D)** for tumor purity.

Supplementary Figure 8 Genomic aberrations in ERGs.

Supplementary Figure 9 USP28 expression comparison.

Supplementary Table 1 Identified prognosis-related eRNA.

Supplementary Table 2 The regression coefficient of each gene in the LASSO cox model.

Supplementary Table 3 The results of GO enrichment analysis.

Supplementary Table 4 The clinicopathologic features of TCGA and CGGA patients.

Supplementary Table 5 The PH test results for the prognostic model.

Supplementary Table 6 The GSEA results in the positive correlative group.

Supplementary Table 7 The GSEA results in the negative correlative group.

Supplementary Table 8 The Cox analysis for all prognostic features.
