# Supplementary figures and images for: Development and Validation of a Novel Prognostic Model for Lower-Grade Glioma Based on Enhancer RNA-Regulated Prognostic Genes
Source: Front Oncol. 2022 Mar 1;12:714338. doi: 10.3389/fonc.2022.714338 (PMC8921558; doi:10.3389/fonc.2022.714338)

A

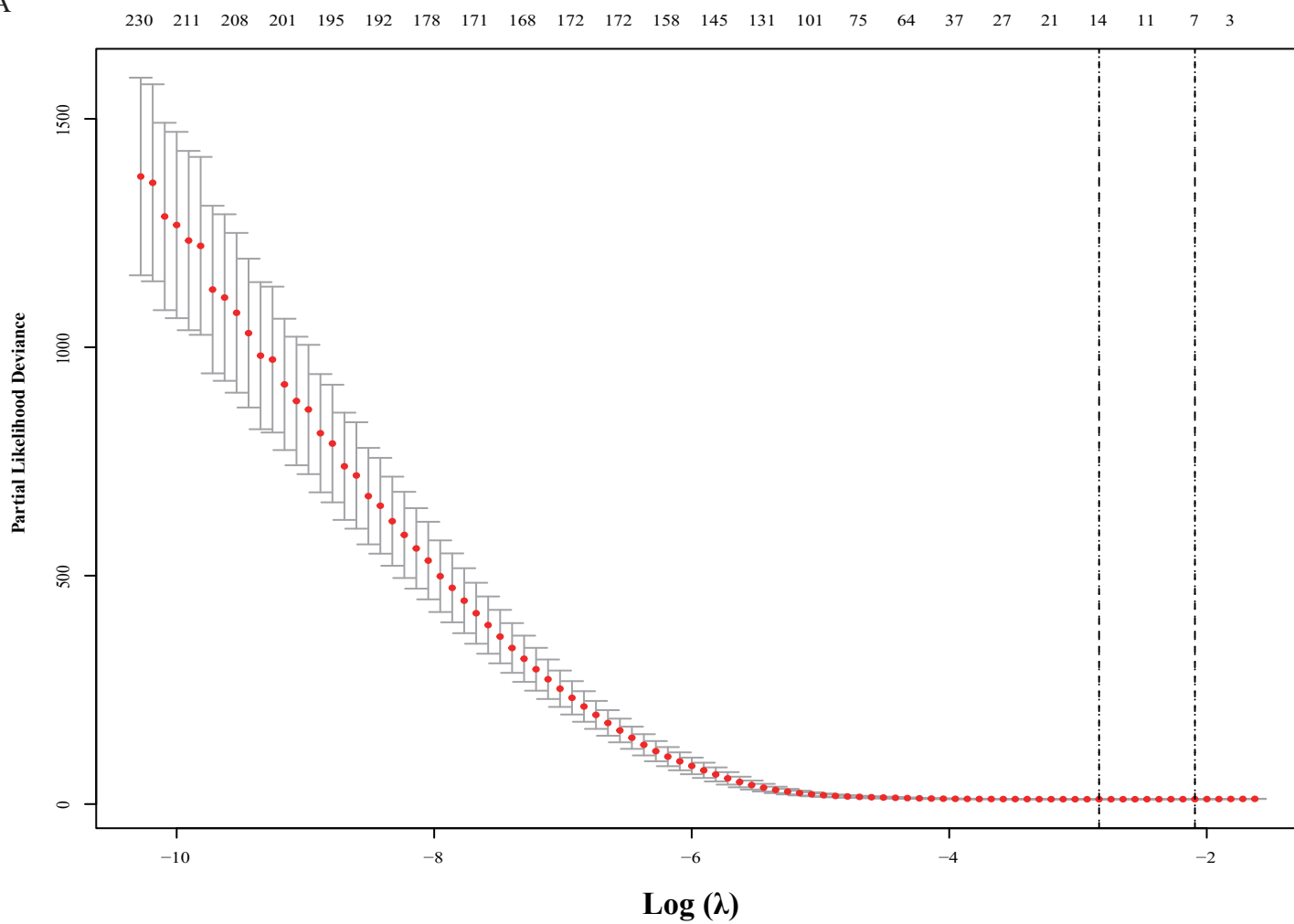

B

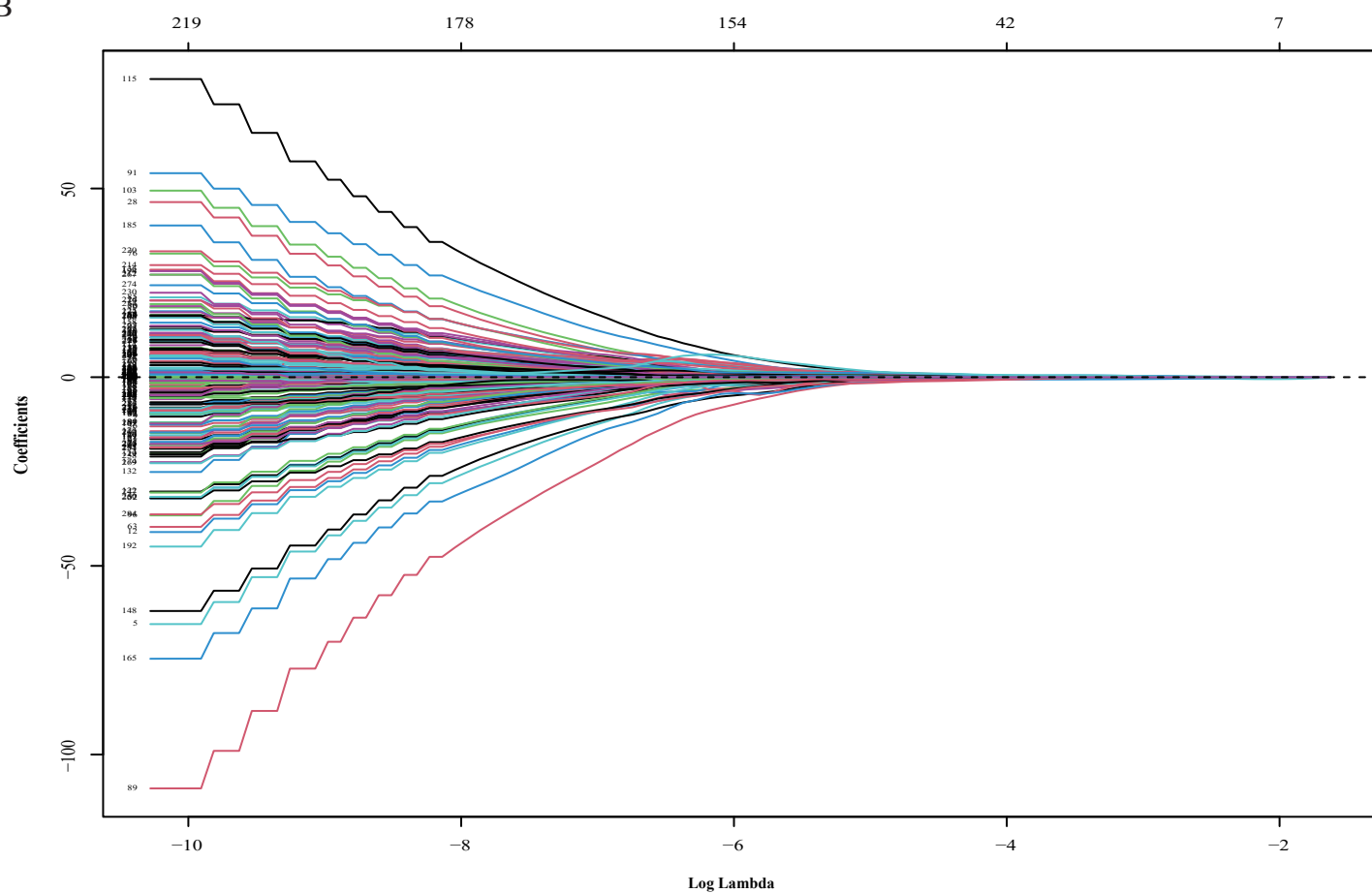

Supplement: Supplementary Figure 1 — The resulting graph of the LASSO cox model. (A) The selection of lambda penalty parameter for the Lasso-Cox model. (B) The coefficient change curve of variables. [file Image_1.pdf]

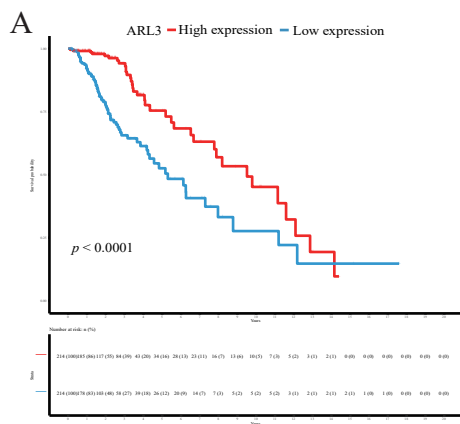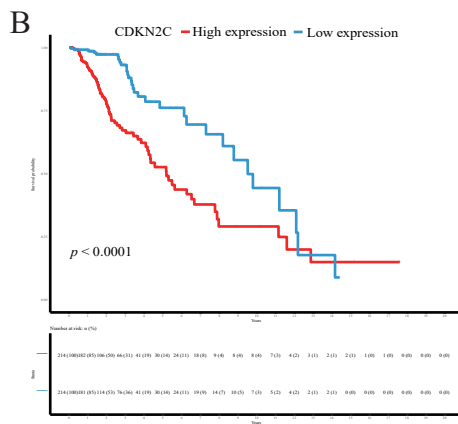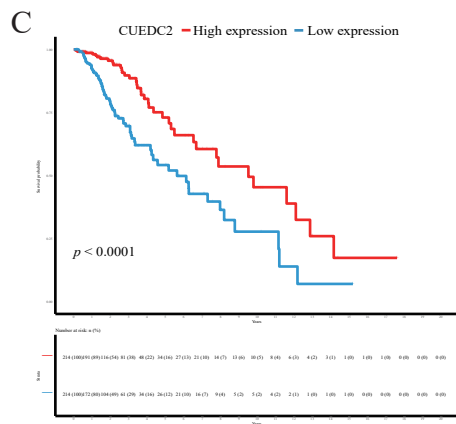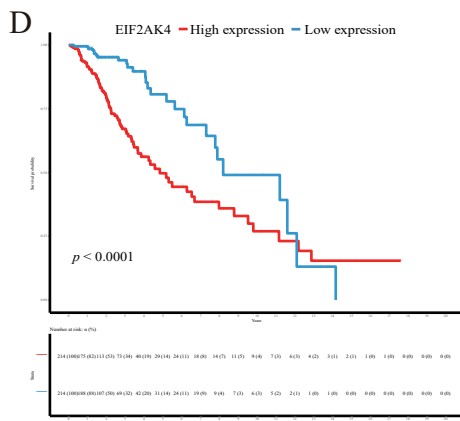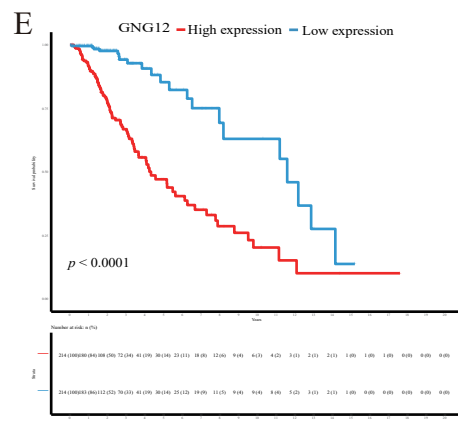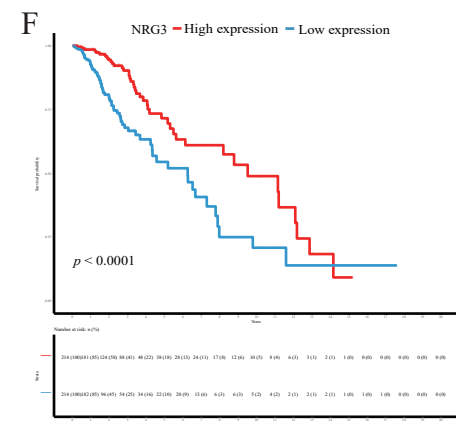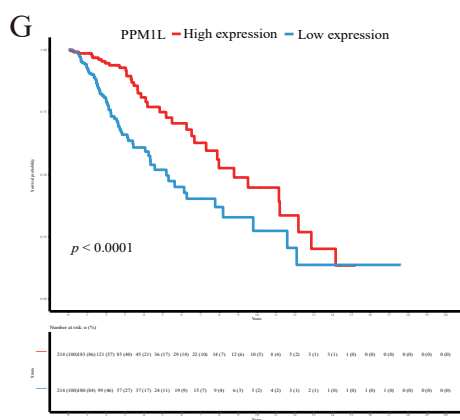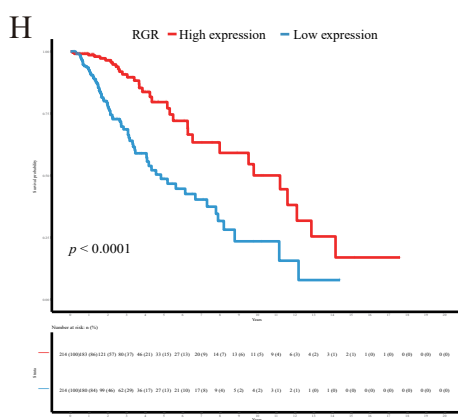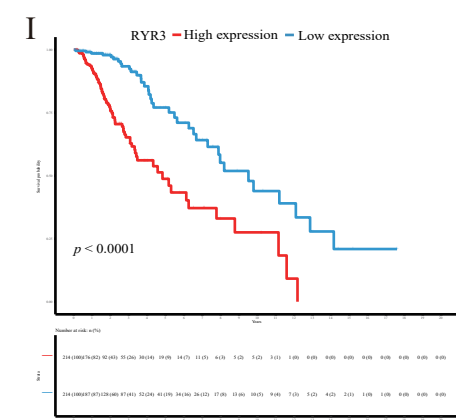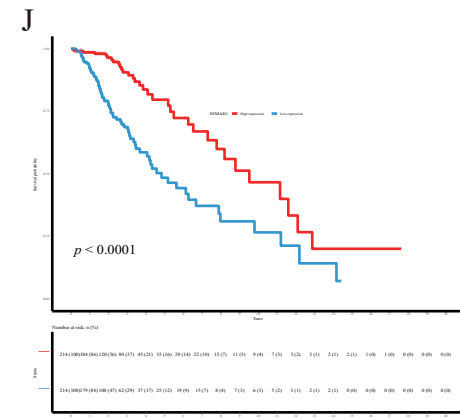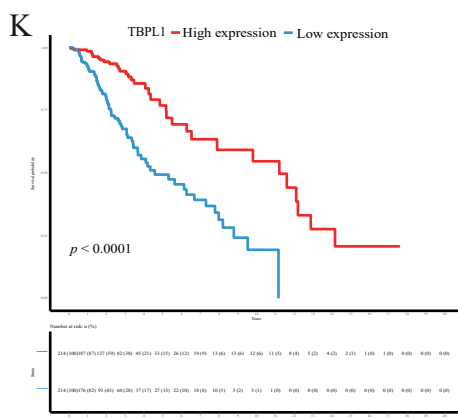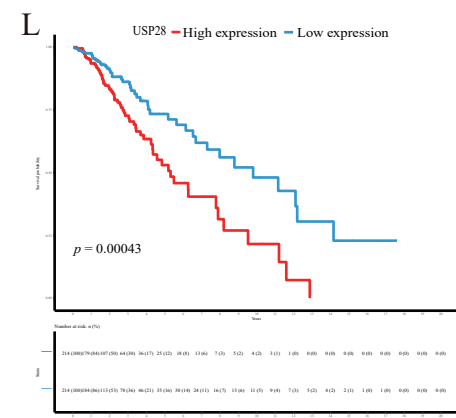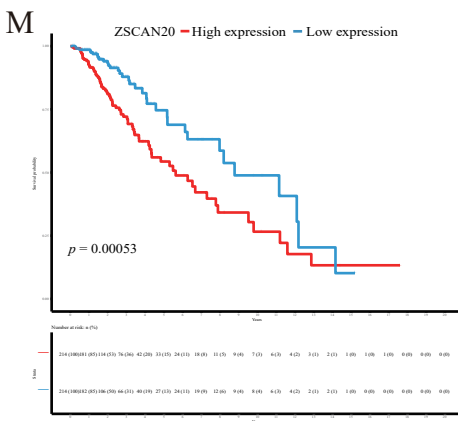

Supplement: Supplementary Figure 2 — The Kaplan-Meier curve for each ERG. [file Image_2.pdf]

IDH ■ Mutant ■ Wildtype

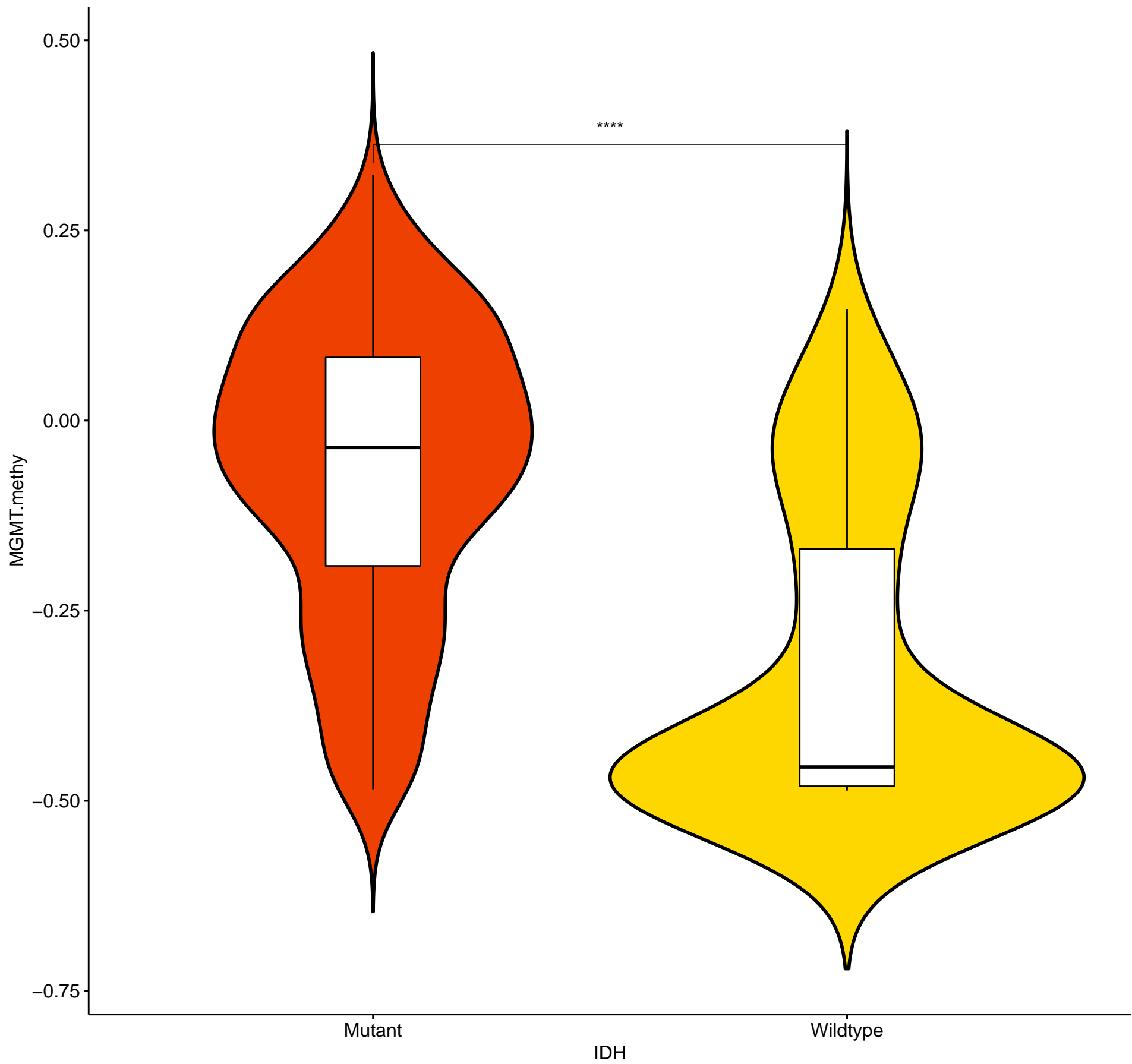

Supplement: Supplementary Figure 3 — The correlation between MGMT methylation and IDH status. [file Image_3.pdf]

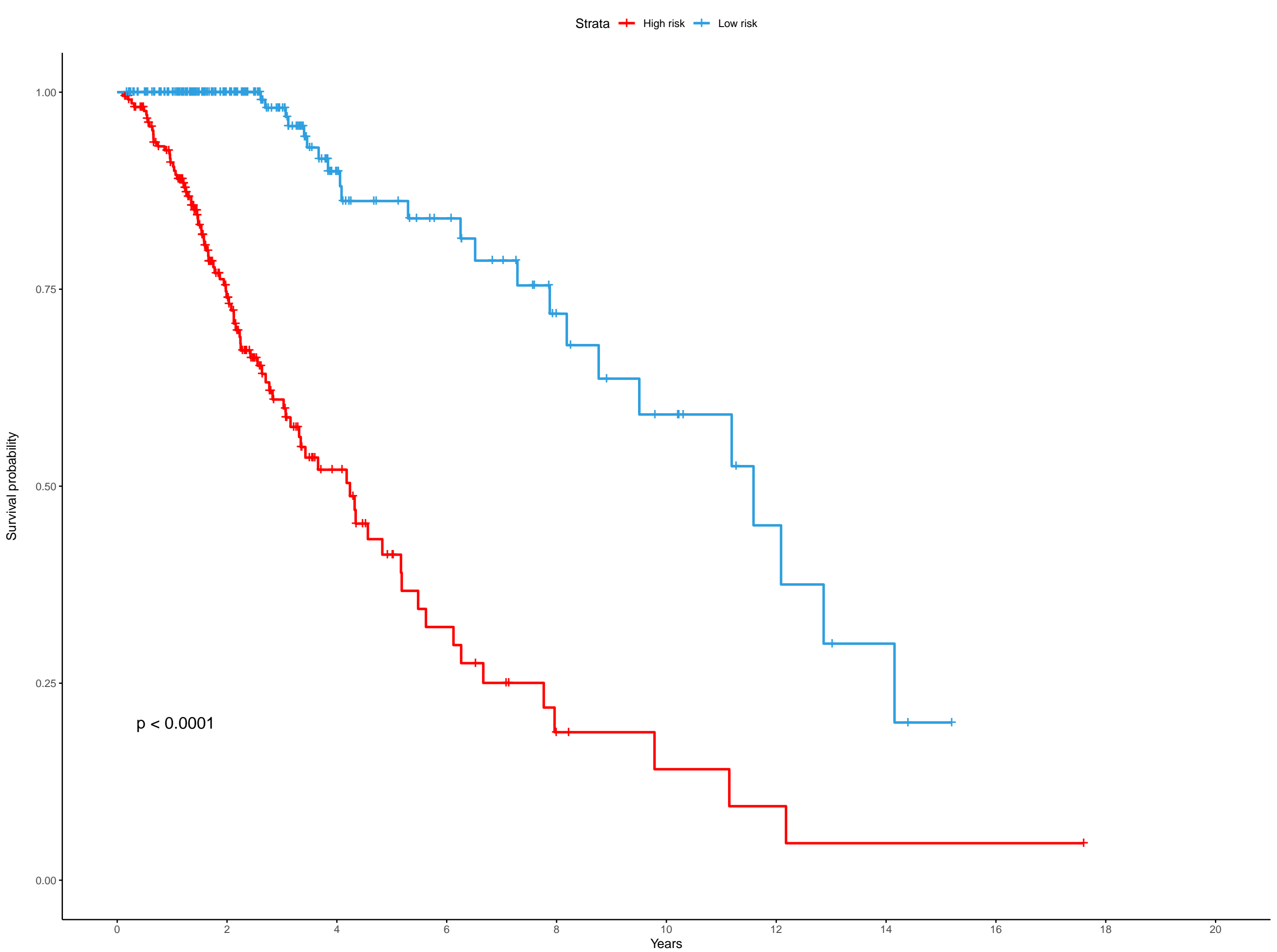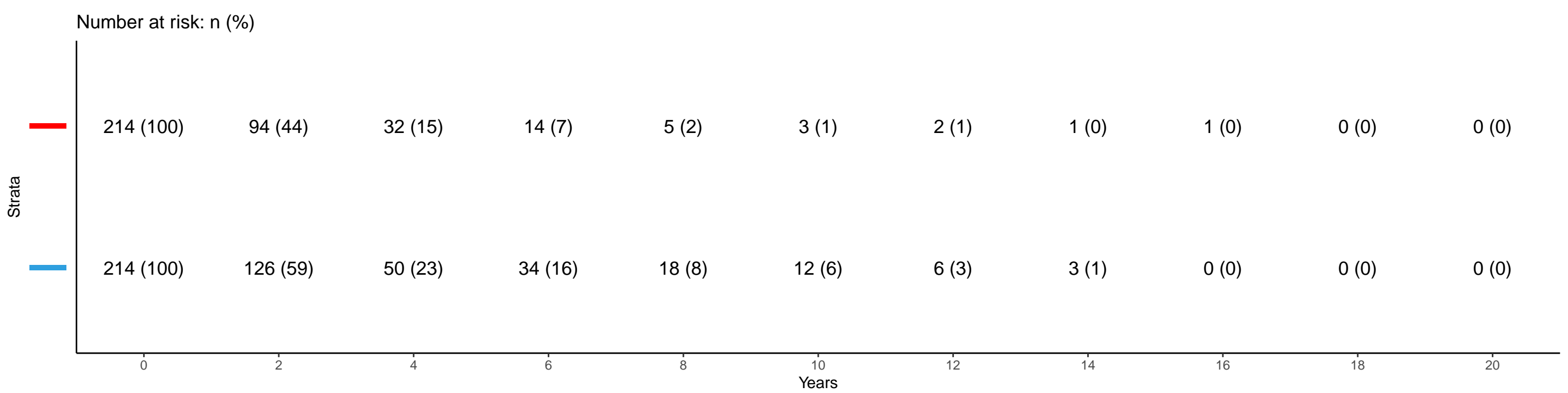

Supplement: Supplementary Figure 5 — The Kaplan-Meier curve for the nomogram in the TCGA cohort. [file Image_5.pdf]

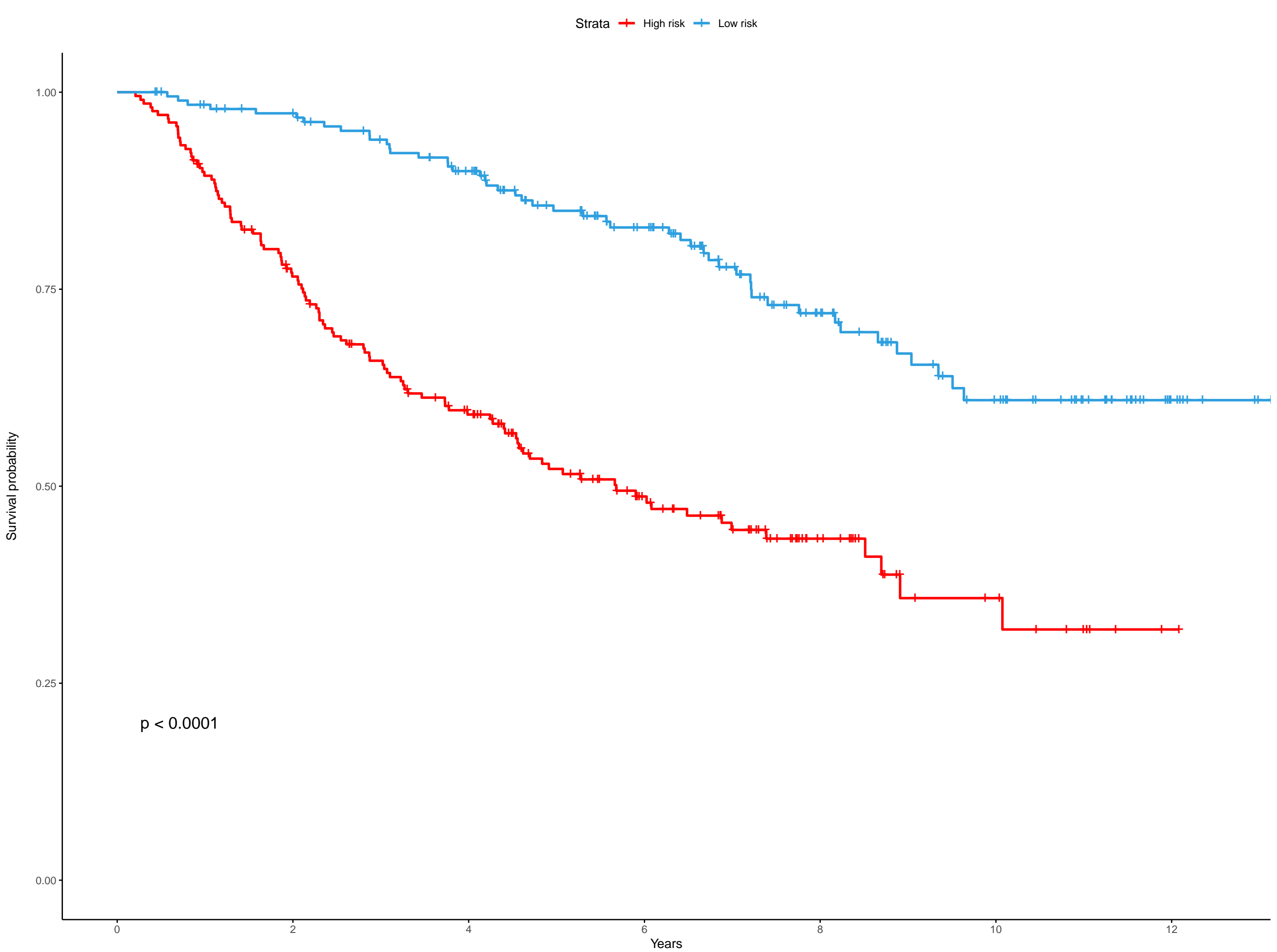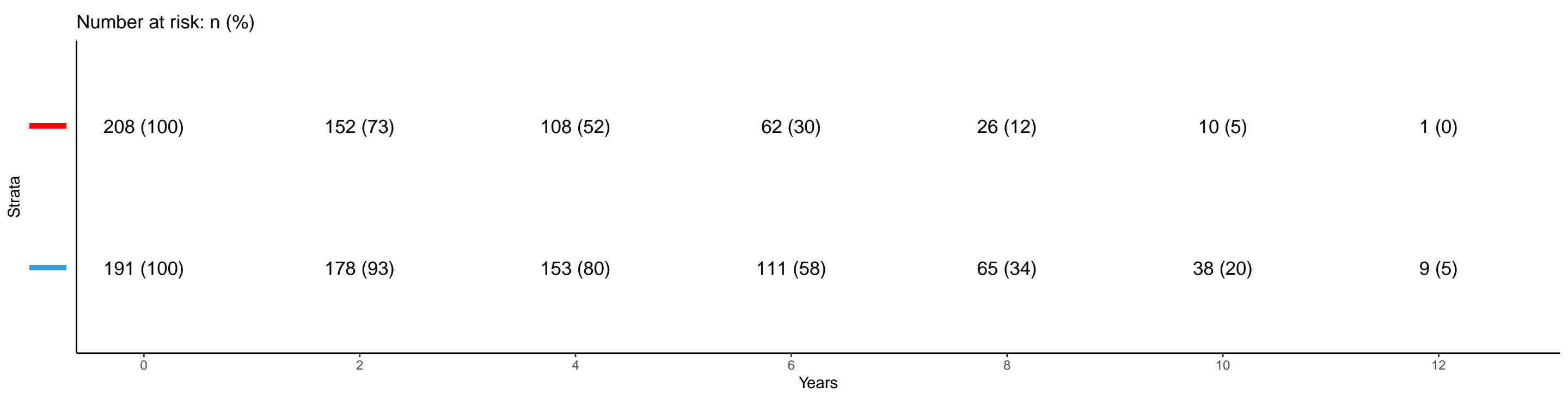

Supplement: Supplementary Figure 6 — The Kaplan-Meier curve for the nomogram in the CGGA cohort. [file Image_6.pdf]

A

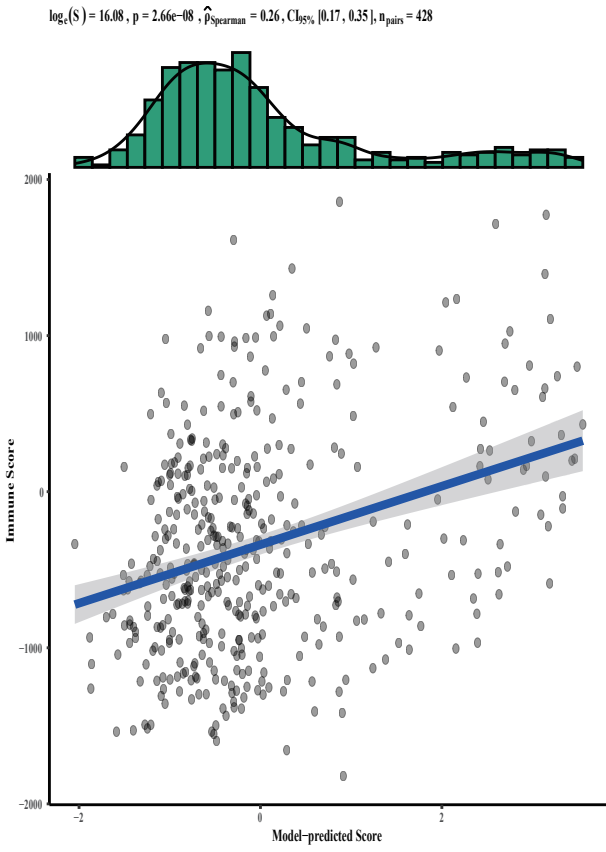

B

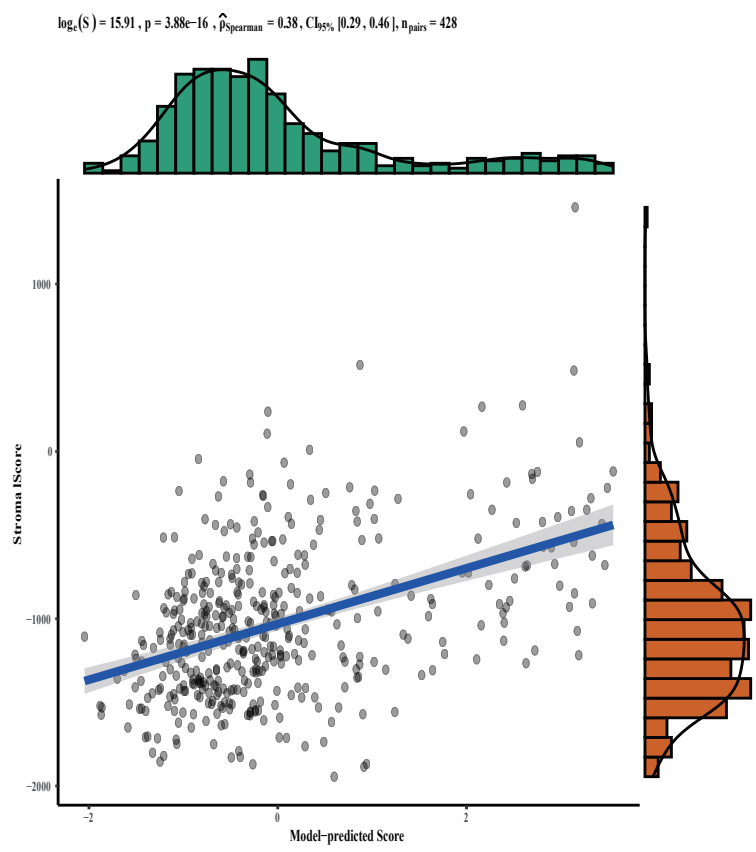

C

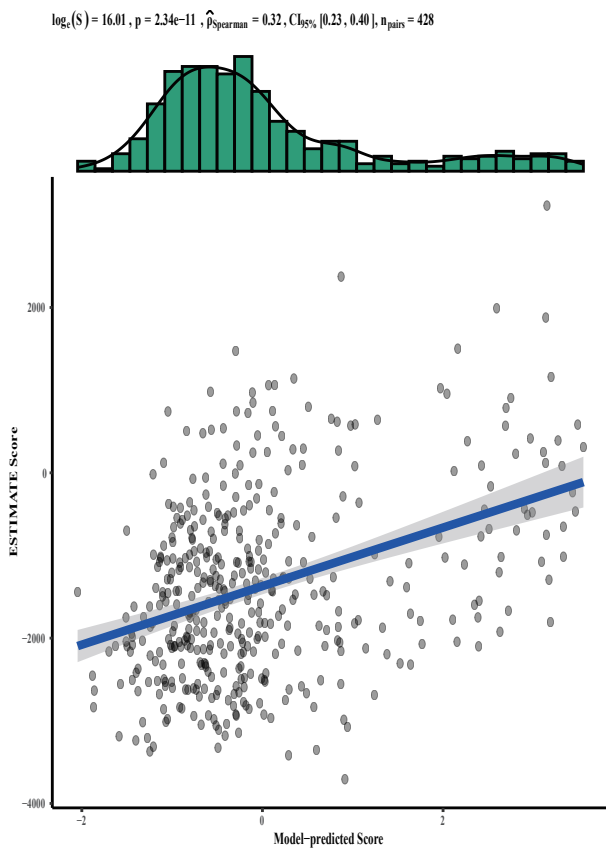

D

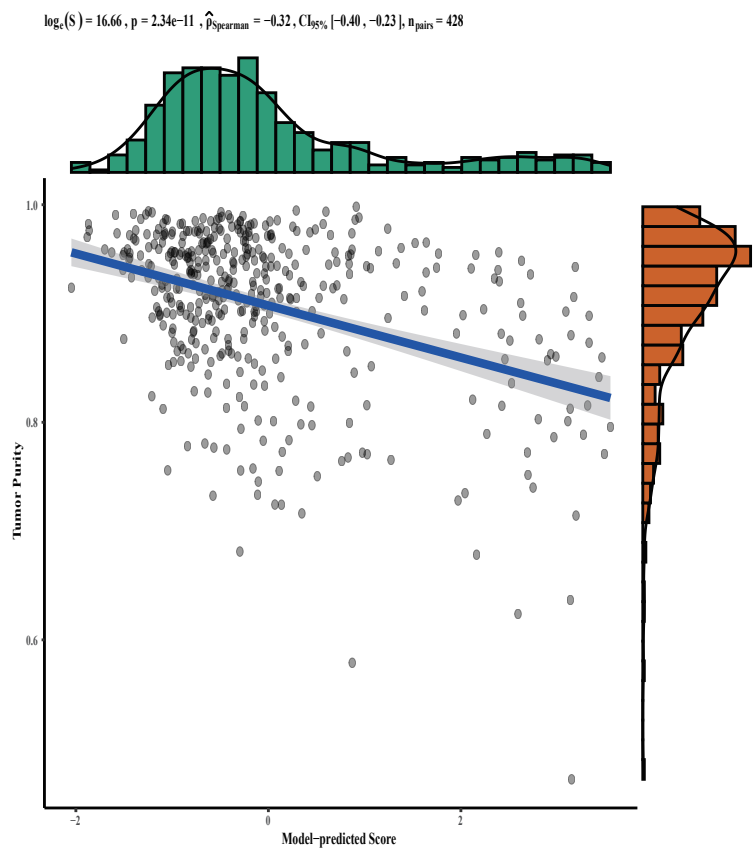

Supplement: Supplementary Figure 7 — The correlation between prognostic model and immune infiltration profiles. (A) correlation analysis for the immune score; (B) for the stromal score; (C) for estimate score; (D) for tumor purity. [file Image_7.pdf]

Altered in 8 (1.68%) of 477 samples.

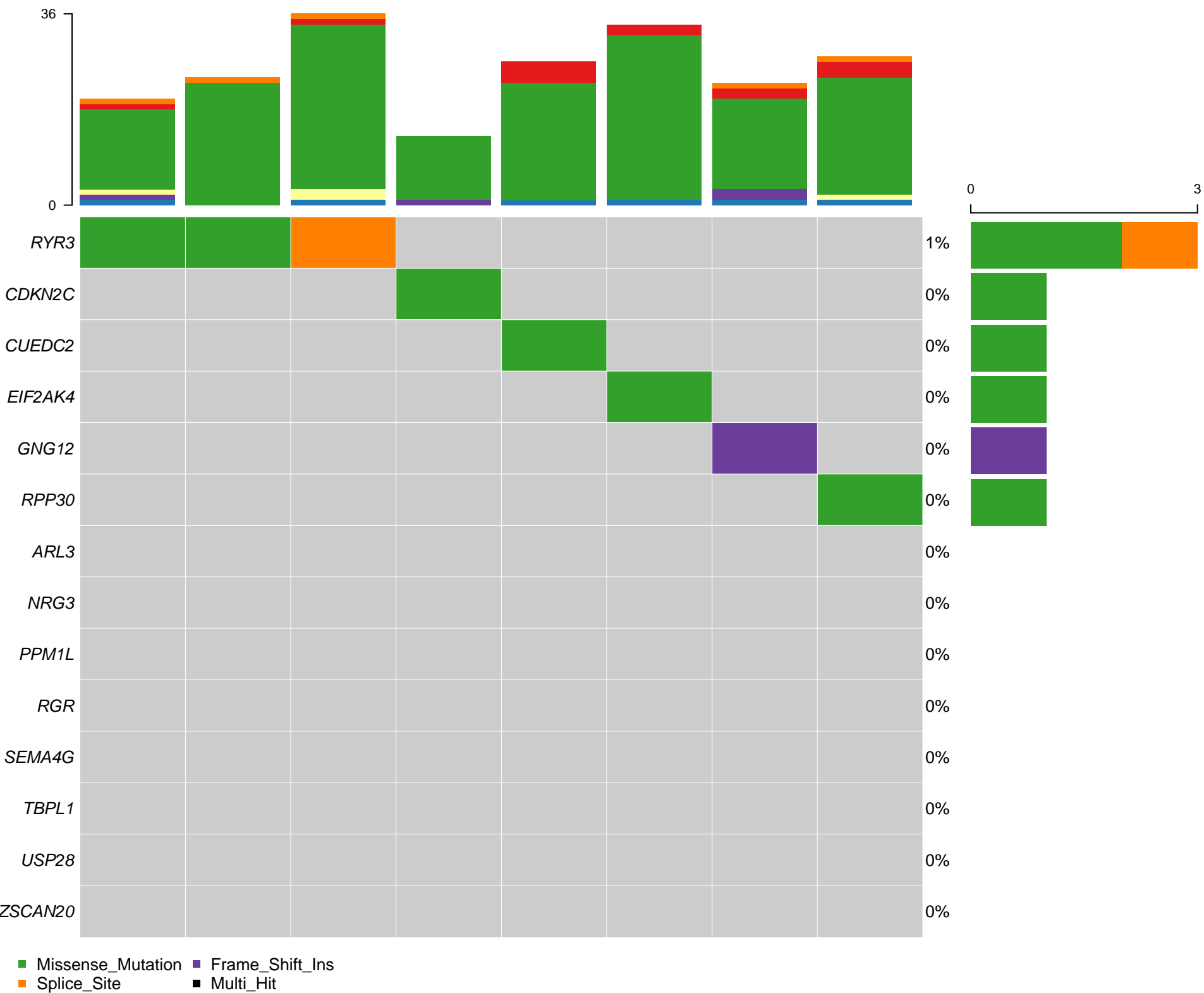

Supplement: Supplementary Figure 8 — Genomic aberrations in ERGs. [file Image_8.pdf]

Sample type 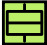 LGGs 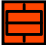 GBM 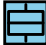 Normal

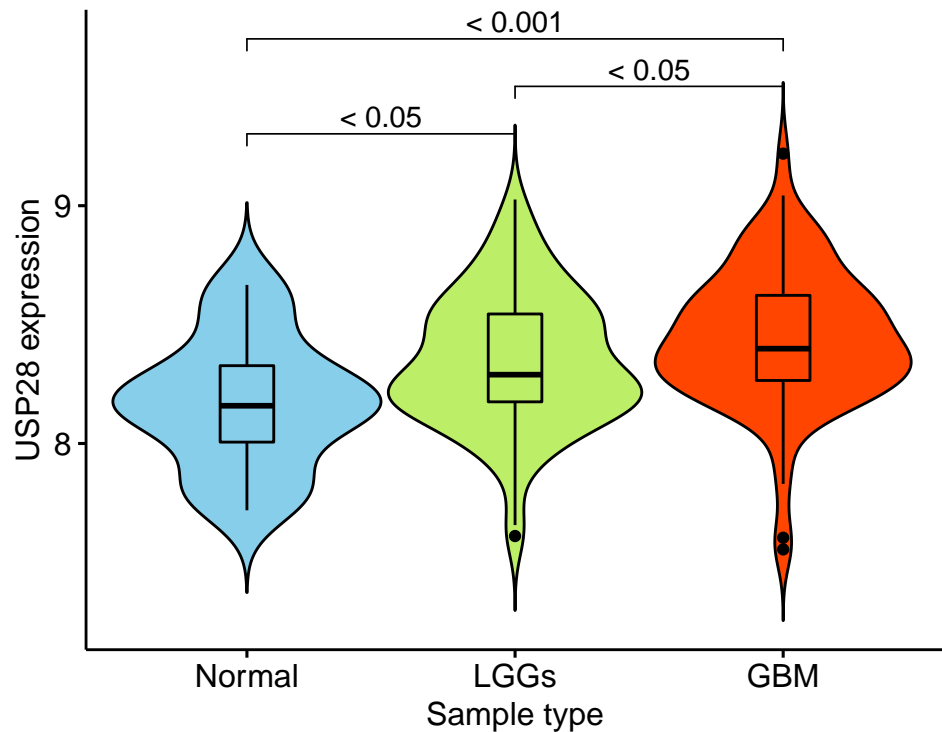

Supplement: Supplementary Figure 9 — USP28 expression comparison. [file Image_9.pdf]
