# Supplementary material for: Development and Validation of a Novel Prognostic Model for Lower-Grade Glioma Based on Enhancer RNA-Regulated Prognostic Genes
Source: Front Oncol. 2022 Mar 1;12:714338. doi: 10.3389/fonc.2022.714338 (PMC8921558; doi:10.3389/fonc.2022.714338)
Supplement: Supplementary Figure 4 — The PH test figure for the prognostic model. [file Image_4.pdf]

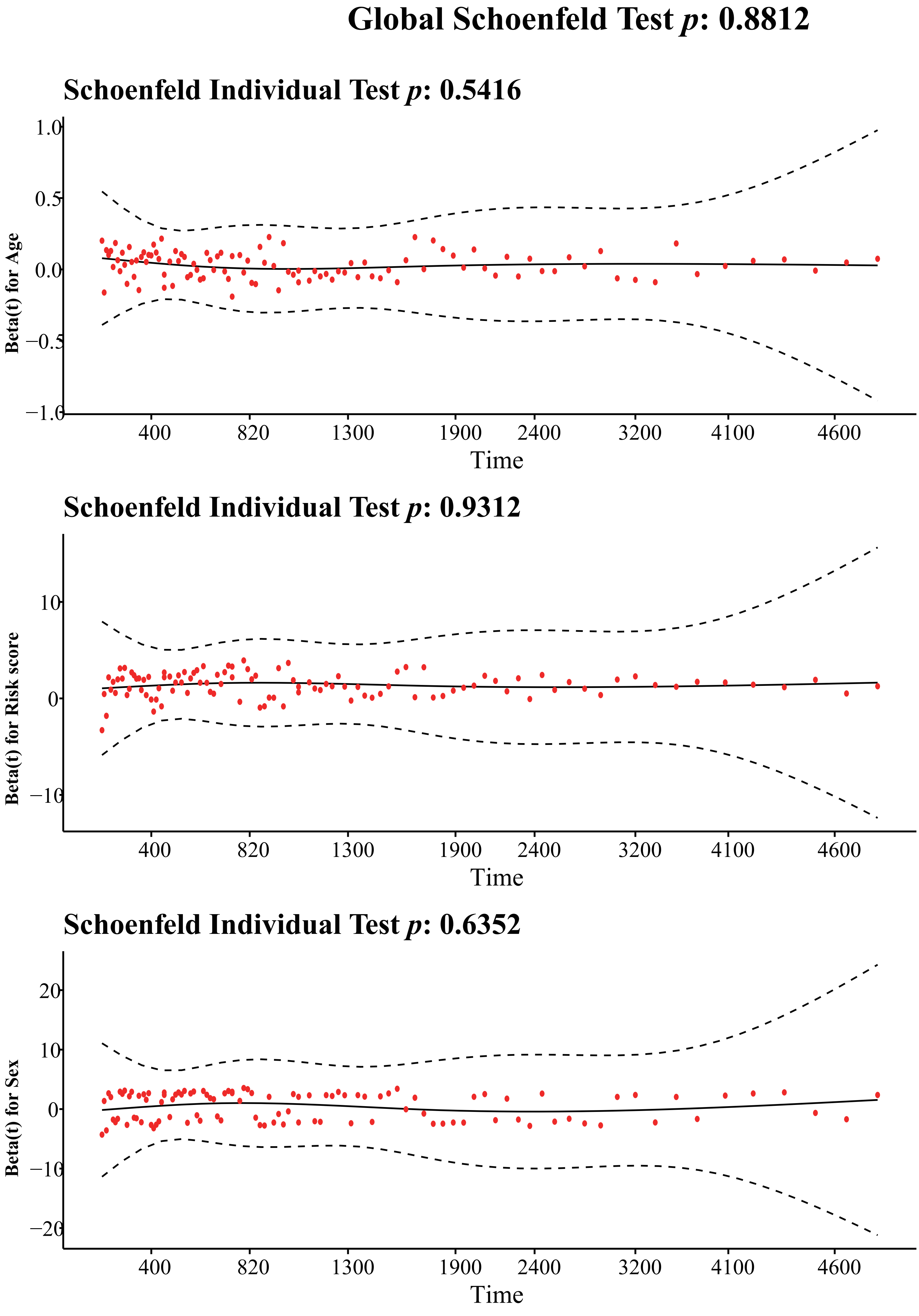
Global Schoenfeld Test p: 0.8812
Schoenfeld Individual Test p: 0.5416
1.0
0.5
0.0
−0.5
−1.0
Beta(t) for Age
400
820
1300
1900
2400
3200
4100
4600
Time
Schoenfeld Individual Test p: 0.9312
10
0
−10
Beta(t) for Risk score
400
820
1300
1900
2400
3200
4100
4600
Time
Schoenfeld Individual Test p: 0.6352
20
10
0
−10
−20
Beta(t) for Sex
400
820
1300
1900
2400
3200
4100
4600
Time
